# Novel Quinoline Compound Derivatives of NSC23925 as Potent Reversal Agents Against P-Glycoprotein-Mediated Multidrug Resistance

**DOI:** 10.3389/fchem.2019.00820

**Published:** 2019-12-19

**Authors:** Xingping Quan, Hongzhi Du, Jingjing Xu, Xiaoying Hou, Xiaofeng Gong, Yao Wu, Yuqi Zhou, Jingwei Jiang, Ligong Lu, Shengtao Yuan, Xiangyu Yang, Lei Shi, Li Sun

**Affiliations:** ^1^Jiangsu Key Laboratory of Drug Screening, China Pharmaceutical University, Nanjing, China; ^2^School of Pharmacy, Hubei University of Chinese Medicine, Wuhan, China; ^3^Henan Key Laboratory of Organic Functional Molecules and Drug Innovation, School of Chemistry and Chemical Engineering, Henan Normal University, Xinxiang, China; ^4^Zhuhai Interventional Medical Center, Zhuhai Precision Medical Center, Zhuhai People's Hospital, Zhuhai Hospital Affiliated with Jinan University, Zhuhai, China

**Keywords:** molecular docking, multidrug resistance (MDR), P-glycoprotein (P-gp), quinoline, reversal cancer resistance

## Abstract

Multidrug resistance is a serious problem and a common cause of cancer treatment failure, leading to patient death. Although numerous reversal resistance inhibitors have been evaluated in preclinical or clinical trials, efficient and low-toxicity reversal agents have not been identified. In this study, a series of novel quinoline compound derivatives from NSC23925 were designed to inhibit P-glycoprotein (P-gp). Among them, YS-7a showed a stronger inhibitory effect against P-gp than verapamil, as a positive control, when co-incubated with chemotherapy drugs at minimally cytotoxic concentrations. YS-7a suppressed the P-gp transport function without affecting the expression of P-gp but stimulated the ATPase activity of P-gp in a dose-dependent manner. Next, molecular docking was used to predict the six most probable binding sites, namely, SER270, VAL273, VAL274, ILE354, VAL357, and PHE390. Moreover, YS-7a had no effect on cytochrome P450 3A4 activity and showed little toxicity to normal cells. In addition, combined treatment of YS-7a with vincristine showed a better inhibitory effect than the positive control verapamil *in vivo* without a negative effect on mouse weight. Overall, our results showed that YS-7a could reverse cancer multidrug resistance through the inhibition of P-gp transport function *in vitro* and *in vivo*, suggesting that YS-7a may be a novel therapeutic agent.

## Introduction

Cancer multidrug resistance (MDR) is a major cause of chemotherapy failure leading to patient death. MDR cancer cells often show pleiotropic cross-resistance to a wide range of chemotherapy drugs. Mechanisms of MDR can be classified into non-cellular-based and cellular-based resistance mechanisms (Krishna and Mayer, 2000). Numerous potential mechanisms of MDR have been reported involving the ABC transporter family, DNA damage and repair, cancer stem cell regulation, microRNA regulation, and epigenetic regulation (Wu et al., 2014). Among these, the ABC transporter families play an important role in cellular-based resistance mechanisms by facilitating exocytosis of chemotherapy drugs (Choi and Yu, 2014; Wu et al., 2014). These transporters are universally expressed across MDR cancer cells, especially P-glycoprotein (P-gp; encoded by MDR1), which is functionally equivalent to an efflux pump that translocate substrates or chemotherapy drugs from the intracellular to the extracellular environment (Fojo et al., 1987; Konstantinos, 2015). Studies have confirmed that P-gp is highly expressed or overactivated in a large number of patients with failed chemotherapy (Alfarouk et al., 2015). Therefore, P-gp is a potential target for reversing drug resistance.

P-gp inhibitors, also known as MDR modulators, have been used to reverse MDR and block P-gp function in combination with chemotherapy drugs (Coley, 2010; Kumar and Jaitak, 2019). Several pharmacological P-gp inhibitors have been developed, including verapamil (VP), PSC-833, and tariquidar. VP was the first to be identified, and is commonly used as a P-gp inhibitor for its low affinity and other pharmacological activities, but has many side effects (Bellamy et al., 1988; Yusa and Tsuruo, 1989). Dexverapamil (Pirker et al., 1995; Thürlimann et al., 1995) and PSC-833 (Boesch et al., 1991; Kusunoki et al., 2010) lack the pharmacological activities of VP and cyclosporin A, but inhibit cytochrome P450 (CYP) 3A4 activity. This leads to complicated drug–drug interactions and limits their application (Chico et al., 2001; Labrie et al., 2006). Meanwhile, tariquidar (XR9576) (Federica et al., 2004; Fox and Bates, 2007), LY335979 (Dantzig et al., 1999; Shepard et al., 2003), and HM30181 (Cha et al., 2013; Köhler and Wiese, 2015) show more specific affinities to P-gp with fewer side effects. Several clinical trials are underway that are expected to address clinical drug resistance. Oraxol, the oral preparation consisting of paclitaxel and HM30181A, showed a strong trend in progression-free survival (*p* = 0.077), favoring oral paclitaxel over *intravenous* paclitaxel and a strong trend in overall survival (*p* = 0.11) (https://ir.athenex.com/, ClinicalTrials.gov Identifier: NCT02594371). However, improved novel inhibitors are required to make P-gp a reliable target for reversing resistance.

NSC23925 was identified from 2,000 small molecule compounds using a high-throughput cell-based screening assay, and can specifically inhibit P-gp and reverse MDR with no effect on P-gp expression (Duan et al., 2009). NSC23925 was shown to prevent the emergence of MDR in ovarian cancer both *in vitro* and *in vivo* (Yang et al., 2015) and in osteosarcoma (Yang et al., 2014) without affecting P-gp expression. NSC23925 also reversed MDR in cancer cells (Duan et al., 2012).

**Figure 1:**
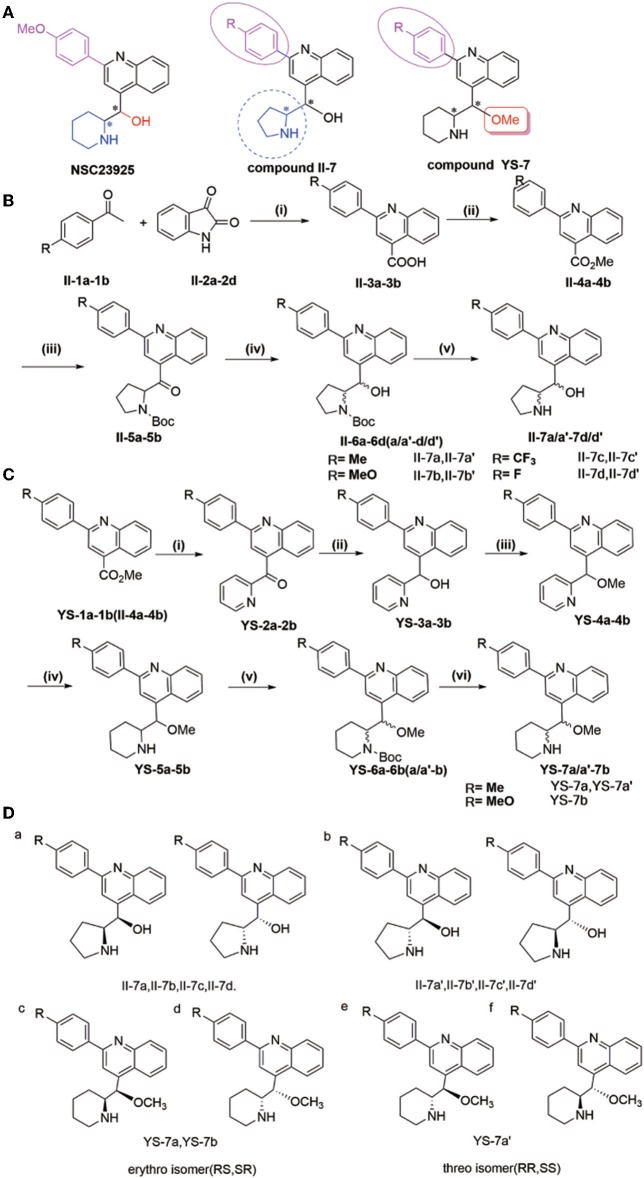
**(A)** Synthesis of compounds II-7a, II-7a′, II-7b, II-7b′, II-7c, II-7c′, II-7d, and II-7d′; **(B)** synthesis of compounds YS-7a, YS-7a′, and YS-7b. Reagents and conditions **(A)**: (i) 1-(4-3-methoxyphenyl)ethanone, KOH, EtOH, 85°C, 24 h; (ii) concentrated H_2_SO_4_, MeOH, 65°C reflux overnight; (iii) 1-Boc-pyrrole (1 equiv), sec-BuLi (1.3 equiv) tetramethylethylenediamine 1.7 mL, −78°C, 2 h, dropping compound, stirring 2 h at room temperature, dry tetrahydrofuran; (iv) NaBH_4_ (9 equiv), EtOH, 0°C, 0.75–1 h. (v) 2 M HCl, 30°C, 48 h. **(B)**: (i) 2-bromopyridine, n-BuLi, Et_2_O/tetrahydrofuran, −78°C, 2 h; (ii) 7 NaBH_4_, EtOH, 0°C, 1 h; (iii) dry N,N-dimethylformamide, NaCl, N_2_, 0°C, 10 min, methyl iodide, stirring 1 h at 25°C; (iv) platinum dioxide, HCl, MeOH, H_2_; (v) triethylamine, di-tert-butyl decarbonate dissolved in tetrahydrofuran at 0°C, stirring at room temperature overnight; (vi) 2 M HCl, 30°C, 48 h. **(C)** Design of target compounds. **(D)** Structure of compounds II-7a, II-7a′, II-7b, II-7b′, II-7c, II-7c′, II-7d, II-7d′, YS-7a, YS-7a′, and YS-7b (a–d).

In this study, we designed and synthesized a series of NSC23925 analogs with improved potency through two mechanisms (Figures 1B,C). Owing to their new structure and superior activity, these compounds were granted a patent (CN 108017615A, CN 107973781A). Among the synthesized compounds, YS-7a and YS-7b showed better P-gp inhibition than the positive control VP and the parent compound NSC23925. Our findings demonstrated that substituting -OH with -OMe increased the intracellular accumulation of Rhodamine 123 (Rho123); therefore, YS-7a was selected for further evaluation of its potent reversal effect. Next, the target of YS-7a was confirmed using small interfering (si)RNA. YS-7a had no effect on mRNA and protein expression of P-gp but inhibited its efflux pumping effect and stimulating P-gp ATPase instead, supporting its direct effect on P-gp. The binding sites were predicted through molecular docking. There was no significant effect on CYP3A4 activity and almost no toxicity toward normal cells. Finally, YS-7a improved the anti-tumor effect of chemotherapy drugs, showing better reversal of drug resistance than VP when combined with vincristine (VCR) *in vivo*. Overall, our study showed that YS-7a inhibited P-gp with high efficiency and low toxicity both *in vitro* and *in vivo*. Therefore, YS-7a is a novel P-gp inhibitor that may be used for the treatment of MDR cancers.

## Materials and Methods

### General Chemistry

All the reagents were obtained from commercial sources and used without further purification unless otherwise indicated. All organic solvents were dried and freshly distilled before use by standard methods. The reactions were monitored by thin layer chromatography (TLC) on GF254 silica gel coated plates and visualized by UV light (254 and 365 nm). Purification by column and flash chromatography was carried out using silica gel (200–300 mesh). Melting points were taken on a X-4B melting-point apparatus and were uncorrected. ^1^H and ^13^C NMR spectra were recorded in DMSO-d6 or CDCl_3_ on a Bruker Avance/600 (^1^H: 600 MHz, ^13^C: 150 MHz at 25°C) or Bruker Avance/400 (^1^H: 400 MHz, ^13^C: 100 MHz at 25°C, Bruker Instruments, Inc., Billerica, MA, USA) Chemical shifts are expressed in values (ppm) relative to tetramethylsilane as an internal standard, and coupling constants (J values) were given in hertz (Hz). Abbreviations are represented as follows: br, broad; s, singlet; d, doublet; dd, double doublet; t, triplet; q, quartet; m, multiplet. HRMS analysis was performed on a mass spectrometer using electrospray ionization (ESI-oa-TOF), and the purity of all samples used for HRMS (>95%) was confirmed by ^1^H and ^13^C NMR spectroscopic analyses. HPLC was performed on Agilent Technologies 1200 LC Column 250 × 4.6 nm and using H_2_O (95–5%)/MeOH (5–95%) during 22 min as the mobile phase. Flow rate was1.0 mL/min (all solvents were HPLC grade). The HPLC system was monitored at 254 nm.

### Biology

#### Cell Lines and Cell Culture

Human leukemia cell line K562, human oral squamous carcinoma KB cells, human hepatocellular carcinoma cell line HepG2 and Human umbilical vein endothelial cells (HUVEC) were obtained from the Cell Bank of the Institute of Biochemistry and Cell Biology, Chinese Academy of Sciences (Shanghai, China).The MDR1-overexpressed cell lines, 3 μM adriamycin (ADR)-selected (Dalian Meilun Biotech Co., Ltd., China) multidrug resistance cell K562/ADR and 0.1 μM vincristine (VCR)-selected (Lingnan Pharmaceutical Co., China) multidrug resistance cell KB/VCR were obtained from Nanjing Shenghe Pharmaceutical Ltd (Nanjing, China). All the cells were cultured in RPMI-1640 medium (Gibco) supplemented with 10% (v/v) heat-inactivated fetal bovine serum (FBS, HyClone) at 37°C and 5% CO_2_.

#### Antibodies and Reagents

The antibodies and reagents included β-actin (AC026, ABclonal), MDR1 (22336-1-AP, proteintech), Anti-rabbit IgG (H+L) (Biotinylated Antibody #14708, Cell Signaling Technology), Anti-mouse IgG (H+L) (Biotinylated Antibody #14709, Cell Signaling Technology).

#### Rhodamine-123 (Rho-123) Accumulation

Intracellular fluorescence intensity was measured by Flow Cytometry (Wang et al., 2000). Cells in the exponential growth phase were seeded in 12-well plates, with about 10^4^ cells per well. After a 24 h incubation at 37°C in a 5% CO_2_, cells were treated with various concentrations of candidate compounds and verapamil (VP) for 4 h. Then 1 μg/mL Rhodamine-123 (Rho-123) was added directly to the cells. Additional incubation for 1 h at 37°C protected from light, the cells were harvested and immediately detected by flow cytometric (BD) at an excitation wavelength of 488 nm and emission wavelength of 530 nm. The results were calculated by GraphPad Prism 6.0 software.

#### Cell Proliferation Assays *in vitro*

The inhibition of the compounds on the growth of cancer cells KB/VCR and KB or human leukemia cell line K562/ADR and K562 cells were estimated by the 3-(4,5-dimethyl-2-thiazolyl)-2,5-diphenyl-2-H-tetrazolium bromide (MTT). Cells were plated in 96-well-plates, with about 1–2 × 10^3^ cells per well. After 24 h, cells were incubated with various concentrations of compounds and verapamil (VP) for 72 h. MTT was added directly to the cells, and incubated for a further 4 h at 37°C protected from light. Finally, the absorbance at 490 nm was read on a microplate reader (Thermo Fisher Scientific). Experiments were conducted in triplicate at least and repeated three times independently. The inhibition rate was calculated as follows (Wei et al., 2019): inhibition rate (%) = (1– absorbance of the treated group/absorbance of the control group) ×100%. The reversing tumor resistance fold (RF) in resistance cancer cells = IC_50_ (concentration at half-maximum inhibition) of single chemotherapeutic drug/IC_50_ of chemotherapeutic drug combined with YS-7a.

#### siRNA Treatment

All siRNA fragments were synthesized by GenePharma (Shanghai, China). Cells in the logarithmic growth phase were seeded in 6-well-plates, with about 5–6 × 10^5^ cells per well. Once the cells attached (often 8 h later), the medium with 10% FBS was replaced by fresh serum-free medium containing different siRNA for 6–8 h. The sequence of siRNA are as follows:

Negative Control 5′-UUCUCCGAACGUGUCACGUTT-3′
5′-ACGUGACACGUUCGGAGAATT-3′
MDR1-homo-824 5′-GACCAGGUAUGCCUAUUAUTT-3′
5′-AUAAUAGGCAUACCUGGUCTT-3′
MDR1-homo-2187 5′-GCGAAGCAGUGGUUCAGGUTT-3′
5′-ACCUGAACCACUGCUUCGCTT-3′
MDR1-homo-3323 5′-CACCCAGGCAAUGAUGUAUTT-3′
5′-AUACAUCAUUGCCUGGGUGTT-3′.

The medium should then be changed to fresh medium with 10% FBS for a further 24 h. Cells were used to evaluate the effectiveness of knockdown and the cytotoxicity effects; methods were the same as cell proliferation assays *in vitro*. The reversing tumor resistance fold (RF) in P-gp knockdown cancer cells = IC_50_ of single chemotherapeutic drug in P-gp knockdown cancer cells/IC_50_ of chemotherapeutic drug combined with YS-7a in P-gp knockdown cancer cells.

#### RT-qPCR Assays

The expression of the relative genes of cells was detected by RT-qPCR as reported (Hou et al., 2018b). The total RNA was isolated with the TRIzol® Reagent (Vazyme) and then reverse transcribed with the HiScript^TM^ QRT SuperMix for qPCR (Vazyme). The mRNA level was measured using the SYBR Green master mix (Vazyme). The β-actin mRNA served as the control. Primer sequences used for qRT-PCR were as follows:

β-actin 5′-GGACTTCGAGCAAGAGATGG-3′ (forward)
5′- AGCACTGTGTTGGCGTACAG-3′ (reverse)
MDR1 5′-GGAGCCTACTTGGTGGCACATAA-3′(forward)
5′-TGGCATAGTCAGGAGCAAATGAAC-3′ (reverse).

#### Western Blot

The expression of the MDR1 protein was analyzed by Western Blot assays. After being treated with compounds, cells were harvested and lysed, the total protein concentrations were consistent according to the BCA kit (Beyotime Biotechnology, China). Protein lysates (20–30 μg protein per lane) were separated by 8% SDS-PAGE, and then the PVDF membranes were incubated with primary antibodies and second antibodies. The results were quantified by image analyzer (Bio-Rad, USA). The expression of β-actin was used as the control.

#### P-gp ATPase Activity Assays

The P-gp ATPase activity was tested by Pgp-Glo™ Assay Systems (Promega). The impact of candidate compounds on P-gp ATPase activity were examined by comparing untreated samples and samples treated with Na_3_VO_4_ (sodium orthovanadate). The compounds could be assessed as stimulating, inhibiting, or having no effect on basal P-gp ATPase activity.

#### Molecular Docking

Schrödinger was used for the molecular modeling studies as we have used before (Hou et al., 2018a). The crystal structure of the P-gp (PDB ID: 3WME) was prepared using Protein Preparation Wizard. The structure of compounds YS-7a were prepared using ChemBioDraw Ultra 13.0. The calculation was performed based on the force field OPLS (optimized potentials for liquid simulations) 2005 selecting water as the solvent. The following structure was obtained from the result of 1,000 calculation cycles.

#### CYP3A4 Activity Assays

The effect of the candidate compounds against CYP3A4 activity was performed by P450-Glo™ CYP3A4 Assay (Luciferin-IPA) Cell-Based/Biochemical Assay (Promega). The expression of the CYP3A4 gene of HepG2 cells could be induced by 25 μM rifampicin. Cells were typically exposed for 24–72 h. The cells were then treated with various concentrations of test compounds for 72 h. Ketoconazole was used as the positive group. Finally, the P450-Glo™ CYP3A4 Assay (Luciferin-IPA) Cell-Based/Biochemical Assay (Promega) was added to pyrolysis, the treated HepG2 cells, and the fluorescence was detected by SoftMax Pro 6.1 (Beckman counter). The relative CYP3A4 activity was estimated as the formula: (the fluorescence of YS-7a treated/YS-7a treated HepG2 cells)/(the fluorescence of control/control HepG2 cells).

#### *In vivo* Experiments

Female BALB/c mice aged 4–6 weeks and weighing 18–20 g were purchased by the Model Animal Research Center of Nanjing University (Nanjing, China). KB/VCR cells (2 × 10^6^) were transplanted to establish the subcutaneous xenograft model. Intraperitoneal administration once the tumor volume (TV) reached 100 mm^3^, all the mice could be divided into six groups: the normal group (saline, 0.2 ml/kg), single chemotherapy drug group (VCR 0.5 mg/kg), positive drug single group (VP 10 mg/kg), single YS-7a group (YS-7a 10 mg/kg), positive drug group (VP 10 mg/kg+ VCR 0.5 mg/kg), and the combination YS-7a group (YS-7a 10 mg/kg + VCR 0.5 mg/kg). All the drugs were given by intraperitoneal injection every 3 days. After 24 days, all the animals were sacrificed and the final tumor obtained to calculate the relative tumor volume (RTV). TV and RTV were calculated as follows: TV (mm^3^) = $\frac{1}{2}$ × A × B^2^, where A represents the longest diameter of tumor, B represents the shortest diameter. RTV = V_24_/V_0_, V_24_ represents the TV of day 24, and V_0_ represents the TV of day 0. Additionally, all of the final tumors were weighted, and the weight loss ratio counted. The weight loss ratio (%) = ${\text{M}}_{\text{24}(\text{drugtreated})}/\text{M}24_{\left(\text{control}\right)}^{*}$100%. In this study, animal administration was guided by the Animal Care and Control Committee of the China Pharmaceutical University.

### Statistical Analysis

All results are shown as the mean ± S.D of triplicate experiments. One-way ANOVA or the student's *t*-test was performed for the statistical analysis using the GraphPad Prism 5.0 software as previously reported (Du et al., 2018). All comparisons are made relative to the untreated controls. ^*^P < 0.05 was considered statistically significant; ^**^P < 0.01 and ^***^P < 0.001 was considered very statistically significant.

## Results

### Design Novel Derivatives With Potent Reversal Activity

The natural compound NSC23925 contains a quinoline structure with one benzene ring and two chiral carbons in the side chain (Figure 1A). Based on the structural mode, we designed and obtained some derivatives **II-7a-a′, II-7b-b′, II-7c-c′, II-7d-d′, YS-7a-a′,** and **YS-7b** by changing the substituent on benzene ring in the side chain and replacing piperidine with tetrahydropyrrole (**compound II-7**) or replacing -OH with -OMe (**compound YS-7**) (Figure 1A). Each compound was purified by column chromatography and the purity of compounds was evaluated using high-performance liquid chromatography (HPLC) (Table S1, Figures S1–S11).

The general procedure for the synthesis of the compounds shown in Figures 1B,C is as follows: 2-(4-R-Phenyl)quinoline-4-carboxylic acid (**II-3**) was prepared from 4-R-1-phenyl ethenone (**II-1**) and indoline-2,3-dione (**II-2**) (yield: 80–82%), and was then esterified to afford methyl-2-(4-R-phenyl)quinoline-4-carboxylate (**II-4** or **YS-1**) (yield: 80–88%). Using n-BuLi as a base, 2-bromopyrrole or 2-bromopyridine was reacted with (**II-4** or **YS-1**) to give (2-(4-R-phenyl)quinolin-4-yl)(pyrrole-2-yl)-methanone (**II-5**) (yield: 20–25%) and (2-(4-R-phenyl)quinolin-4-yl)(pyridin-2-yl)-methanone (**YS-2**) (yield: 80–85%). Subsequent reduction of **II-5** or **YS-2** by NaBH_4_ afforded 2-(4-R-phenyl) (quinolin-4-yl)(pyridin-2-yl)methanol (**II-6**) (yield: 87–96%) and 2-(4-R-phenyl) (quinolin-4-yl)(pyridin-2-yl)methanol (**YS-3**) (yield: 90–98%). **II-6** was separated by column chromatography to obtain a pair of diastereomers **II-6** (**a, a′-d, d′**) that could be separated based on polarity; the less polar compound was named **II-6** (**a-d**) and the more polar was named **II-6** (**a′-d′**). Methyl iodide was used as a methyl donor to produce methyl-2-(4-R-phenyl) (quinolin-4-yl)(pyridin-2-yl)methyl ether (**YS-4**) (yield: 85%). We then performed a reduction reaction of **YS-4** by hydrogen to produce 2-(hydroxy(2-(4-R-phenyl) quinolin-4-yl) methyl) piperidine-1-carboxylic acid tert-butyl ester (**YS-5**) (yield: 95%). A triethylamine and di-tert-butyl decarbonate reaction with **YS-5** was used to produce **YS-6**, after which **YS-6** was separated by column chromatography to obtain a pair of diastereomers that could be separated based on polarity; the less polar compound was named **YS-6** (**a** or **b**) and the more polar was named **YS-6** (**a′** or **b′**) (yield: 75%). The deprotection reaction of **II-6** or **YS-6** was performed and finally solid washed with dichloromethane to yield the corresponding diastereomer **II-7** (**a, a′-d, d′**) and **YS-7** (**a, a′** or **b**).

Based on a previous study of the Erythro/Threo configuration of phenyl 2-piperidylcarbinols by NMR, after knowing its absolute configuration (Lapidus and Fauley, 1971; Solladié-Cavallo et al., 2003), the ^3^J-erythro and ^3^J-threo in the CH(OH)-CH(NH) systems were different, always showing ^3^J-erythro(RS, SR) < ^3^J-threo(RR, SS). Therefore, ^1^H NMR was applied to determine the structure of these compounds. By analyzing these data, we found that the low polarity compounds (II-7a,7b,7c) could belong to etythro (RS, SR) isomer, and the highly polar compounds (II-7a',7b',7c') belong to threo (RR, SS) isomer. Therefore, we inferred that YS-7a and YS-7b (the lower polarity) belongs to etythro (RS, SR) isomer compounds, and YS-7a' belongs to the threo (RR, SS) isomer compound (Figure 1D). All NMR spectra of these compounds are provided in Figures S12–S55. After obtaining these 11 compounds, the reversal activities were measured.

### Screening of Novel P-gp Inhibitors in MDR Cells

**Figure 2:**
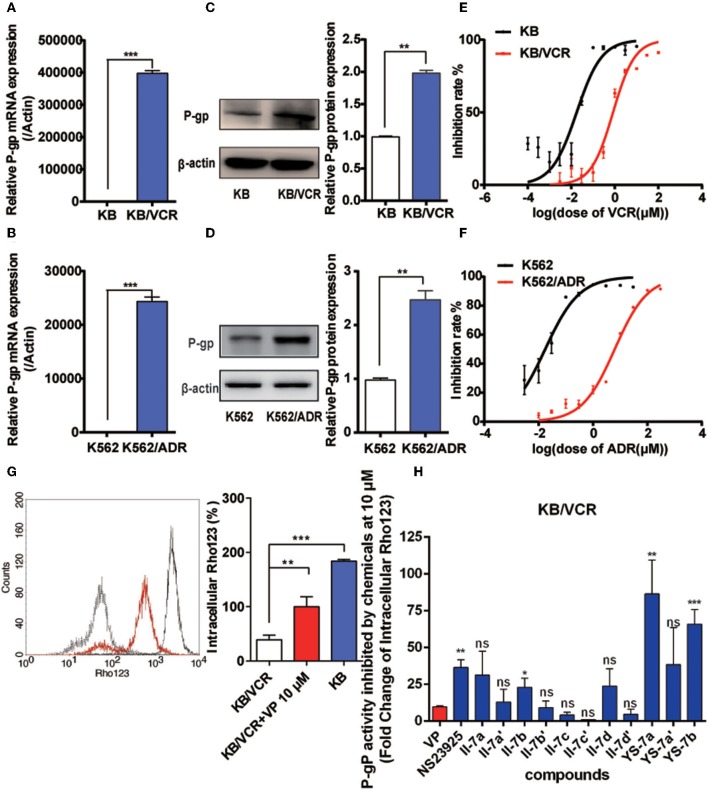
Drug resistance of the KB/VCR and K562/ADR cell lines and screening for novel P-gp inhibitors. **(A,B)** Expression of P-gp mRNA in KB, KB/VCR, K562, and K562/ADR cells. **(C,D)** Expression of P-gp protein in KB, KB/VCR, K562, and K562/ADR cells. **(E,F)** The IC_50_ was determined after exposure to a series concentration of chemotherapy drugs (VCR or ADR) in KB, KB/VCR, K562, and K562/ADR cells for 72 h, and were measured using the MTT assay. **(G)** Intracellular content of Rho123 in KB cells, KB/VCR cells, and KB/VCR cells exposed to 10 μM VP for 4 h. **(H)** Flow cytometry was used to screen compounds that exhibited strong P-gp inhibition after incubation for 4 h; YS-7a and YS-7b resulted in more than 50 times accumulation of Rho123, and were chosen for further study. All experiments were repeated at least three times. **P < 0.01 and ***P < 0.001.

We evaluated the cancer MDR reversal activities of the candidate compounds. First, quantitative reverse transcription PCR and Western blotting were performed to characterize the resistance of the KB/VCR and K562/ADR cell lines (Figures 2A–F). P-gp expression was increased at both the mRNA and protein levels. MDR of KB/VCR and K562/ADR cells was detected based on the half-maximal inhibitory concentration (IC_50_) using the MTT assay. KB/VCR cells (IC_50_ = 0.8294 ± 0.241 μM) showed a 46.8-fold greater resistance to VCR than KB cells (IC_50_ = 0.01770 ± 0.094 μM), whereas K562/ADR cells (IC_50_ = 6.919 ± 0.01992 μM) showed a 347.3-fold greater resistance to adriamycin (ADR) than K562 cells (IC_50_ = 0.01992 ± 0.008 μM). Because the resistance of cancer cells decreased in the absence of chemotherapy drugs, compounds at a dose below the 20% inhibition concentration (0.1 μM VCR in KB/VCR cells; 3 μM ADR in K562/ADR cells) were co-incubated during the entire process. These findings supported the use of KB/VCR and K562/ADR cells for subsequent experimentation.

After confirming the overexpression of P-gp in MDR cancer cells, we detected the intracellular accumulation of Rho123 to screen for potential novel P-gp inhibitors and to assess P-gp inhibition. Rho123 was added to KB and KB/VCR cells at a final concentration of 5 μM for 1 h; 10 μM VP in KB/VCR cells were used as the positive control. The intracellular concentration of Rho123 decreased significantly in KB/VCR cells, which was reversed by VP (Figure 2G). Subsequently, II-7a, II-7a′, II-7b, II-7b′, II-7c, II-7c′, II-7d, II-7d′, YS-7a, YS-7a′, and YS-7b were evaluated by flow cytometry for their capacity to inhibit Rho123 efflux. Among the candidate compounds, the low-polarity compounds YS-7a (fold change = 82.19 ± 17.79) and YS-7b (65.85 ± 10.04) showed the greatest degree of Rho123 efflux inhibition, compared to VP (9.79 ± 0.70) and NSC23925 (40.53 ± 0.49) (Figure 2H), suggesting that they can potently reverse MDR. Further characterization was performed to measure their reversal efficiency *in vitro*.

### Structure–Activity Relationship

Next, we investigated the structure–activity relationship (SAR) of the candidate compounds (Table 1). The low-polarity compound **II-7b**, which substituted piperidine for tetrahydropyrrole, showed significantly decreased activity compared to the parent compound. Changing the substituent on the benzene ring in the side chain with an electron-donating or electron-withdrawing group resulted in compounds **II-7a-a′** (R_1_: -Me), **II-7c-c′** (R_1_: -CF_3_), and **II-7d-d′** (R_1_: -F); these compounds did not improve the efflux of intracellular Rho123. Among these eight compounds (i.e., **II-7a-a′ to d-d′**), higher polarity damaged the activity.

**Table 1:** SAR study of novel quinoline compounds.

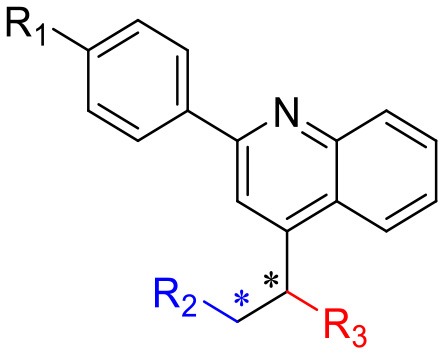				**Structure**	**tR(min) /HPLC**	**Fold change of intracellular Rho123 (FC)**
**Compound**	**-R**_**1**_	**-R**_**2**_	**-R**_**3**_			
**NSC23925**	-OMe	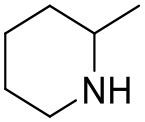	-OH			36.50 ± 5.20
**II-7a**	-Me	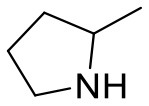	-OH	Etythro (RS, SR) isomer	tR = 19.23 min 96.61%	31.35 ± 16.05
**II-7a′**				Threo (RR, SS) isomer	tR = 19.718 min 95.66%	12.94 ± 8.72
**II-7b**	-OMe			Etythro (RS, SR) isomer	tR = 19.068 min 96.17%	23 ± 6.13
**II-7b′**				Threo (RR, SS) isomer	tR = 19.488 min 95.10%	9.16 ± 4.66
**II-7c**	-CF_3_			Etythro (RS, SR) isomer	tR = 10.917 min 97.35%	4.15 ± 1.96
**II-7c′**				Threo (RR, SS) isomer	tR = 13.772 min 95.03%	1.04 ± 0.01
**II-7d**	-F			Etythro (RS, SR) isomer	tR = 9.931 min 95.70%	23.79 ± 11.86
**II-7d′**				Threo (RR, SS) isomer	tR = 12.429 min 95.00%	4.63 ± 3.57
**YS-7a**	-Me	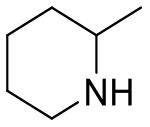	-OMe	Etythro (RS, SR) isomer	tR = 18.222 min 96.33%	86.43 ± 22.92
**YS-7-a′**				Threo (RR, SS) isomer	tR = 19.923 min 96.93%	38.42 ± 25.09
**YS-7b**	-OMe			Etythro (RS, SR) isomer	tR = 19.409 min 97.22%	65.85 ± 10.04

Based on these results, we focused on the replacement of -OH with -OMe in the chiral carbons. The addition of a methoxy group in chiral carbons (**YS-7b**) promoted the activity, suggesting that reducing the polarity of the molecule and hydrogen bonding are beneficial to its activity. Interestingly, **YS-7a**, with an electron-donating methyl substitution, was more potent (fold change = 82.19 ± 17.79) than NSC23925, whereas the high-polarity compound **YS-7a′** (38.42 ± 25.09) showed no obvious improvement in activity. Considering the bioactivity and polarity of the compounds, we selected compounds **YS-7a** and **YS-7b** for further characterization.

### *In vitro* Drug Resistance Reversal Effects of YS-7a and YS-7b

**Figure 3:**
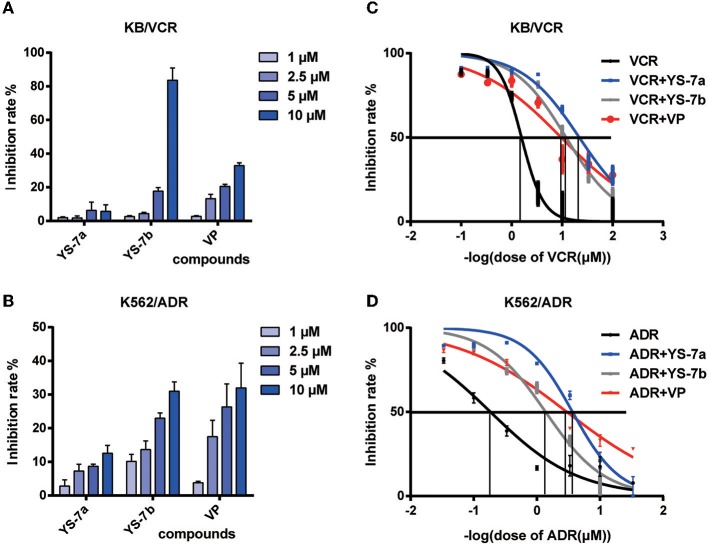
Cytotoxicity and reversal effect of the candidate compounds YS-7a and YS-7b in KB/VCR and K562/ADR cells. The compounds were diluted to four concentrations, 1, 2.5, 5, and 10 μM, and cytotoxicity was evaluated after 72 h using the MTT assay in **(A)** KB/VCR and **(B)** K562/ADR cells. **(C)** Combined treatment of 10 μM YS-7a and 2.5 μM YS-7b with a series of VCR concentrations in KB/VCR cells for 72 h. **(D)** Combined treatment of 10 μM YS-7a and 2.5 μM YS-7b with a series of ADR concentrations in K562/ADR cells for 72 h. The data shown represent the mean ± SD of three independent experiments.

As potential P-gp inhibitors, YS-7a and YS-7b may reverse the resistance to chemotherapy drugs. Before testing YS-7a and YS-7b in combination with chemotherapy drugs, the antiproliferation effects of YS-7a and YS-7b were measured using the MTT assay. A concentration with low antiproliferation effects (<20% inhibition) was used to evaluate their MDR reversal effects (Figures 3A,B). In both KB/VCR and K562/ADR cells, 10 μM YS-7a, 2.5 μM YS-7b, and 2.5 μM VP were selected as the low-toxicity dose.

The drug resistance reversal effects of YS-7a and YS-7b were measured in combination with chemotherapy drugs based on cytotoxicity. Series concentrations of the chemotherapy drugs VCR and doxorubicin (ADR) were applied individually to explore the reversal effects of YS-7a and YS-7b. YS-7a exerted powerful reversal activity compared to the classical P-gp inhibitor VP in both KB/VCR and K562/ADR cells (Figures 3C,D). The IC_50_ values of the combined YS-7a + chemotherapy drug in drug-resistant KB/VCR and K562/ADR cells were 0.0376 ± 0.0116 μM and 0.268 ± 0.053 μM, respectively, whereas the IC_50_ values of the chemotherapy drugs in drug-sensitive KB and K562 cells were 0.435 ± 0.286 μM and 6.571 ± 1.758 μM, respectively; therefore, YS-7a showed a 10.11 ± 3.51 and 30.59 ± 5.83 reversal of drug resistance in KB/VCR and K562/ADR cells, respectively. Meanwhile, the IC_50_ values of the combined YS-7b + chemotherapy drug in drug-resistant KB/VCR and K562/ADR cells were 0.0561 ± 0.0390 μM and 0.616 ± 0.185 μM, respectively, whereas the IC_50_ values of the chemotherapy drugs in drug-sensitive KB and K562 cells were 0.375 ± 0.246 μM and 6.571 ± 1.758 μM, respectively; therefore, YS-7b showed a 6.92 ± 0.55 and 10.79 ± 0.87 reversal of drug resistance in KB/VCR and K562/ADR cells, respectively. Overall, YS-7a showed a significantly better drug resistance reversal effect in MDR cells and was selected for further experimentation of its reversal capabilities.

### P-gp May Be a Target of YS-7a

**Figure 4:**
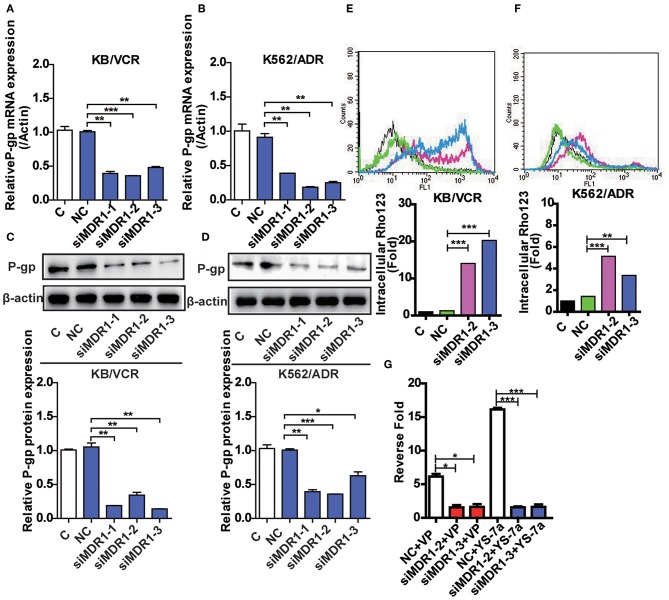
Confirmation of the target of YS-7a in MDR cells. The expression of P-45 gp mRNA and protein in *MDR1* knockdown **(A,C)** KB/VCR cells and **(B,D)** K562/ADR cells. Changes in P-gp transporter function in *MDR1* knockdown **(E)** KB/VCR cells and **(F)** K562/ADR cells. **(G)** The target of YS-7a was verified through the fold change in drug resistance reversal in KB/VCR cells using the MTT assay after *MDR1* knockdown. All results were repeated at least three times. *P < 0.05, **P < 0.01, and ***P < 0.001.

Although YS-7a was screened for its potent reversal effect in MDR cancer cells, it remained unclear whether it targeted P-gp. To clarify this, knockdown of P-gp was implemented by three siMDR1 fragments. siMDR1-2 and siMDR1-3 were chosen for further experiments, both of which downregulated P-gp mRNA and protein levels in KB/VCR and K562/ADR cells (Figures 4A–D). Knockdown by siMDR1-2 and siMDR1-3 decreased P-gp transporter function (Figures 4E,F). After knockdown of P-gp, the drug resistance reversal potency of YS-7a decreased from 16.12- to 1.57-fold (siMDR1-2) and 1.63-fold (siMDR1-3) in KB/VCR cells, whereas that of VP decreased from 6.26- to 1.56-fold (siMDR1-2) and 1.63-fold (siMDR1-3) (Figure 4G). In other words, the reversal effect of YS-7a almost entirely disappeared after P-gp knockdown. Altogether, these results show that the drug resistance reversal effect of YS-7a relies on P-gp.

### YS-7a Does Not Affect P-gp Expression but Directly Inhibits Transport Function

**Figure 5:**
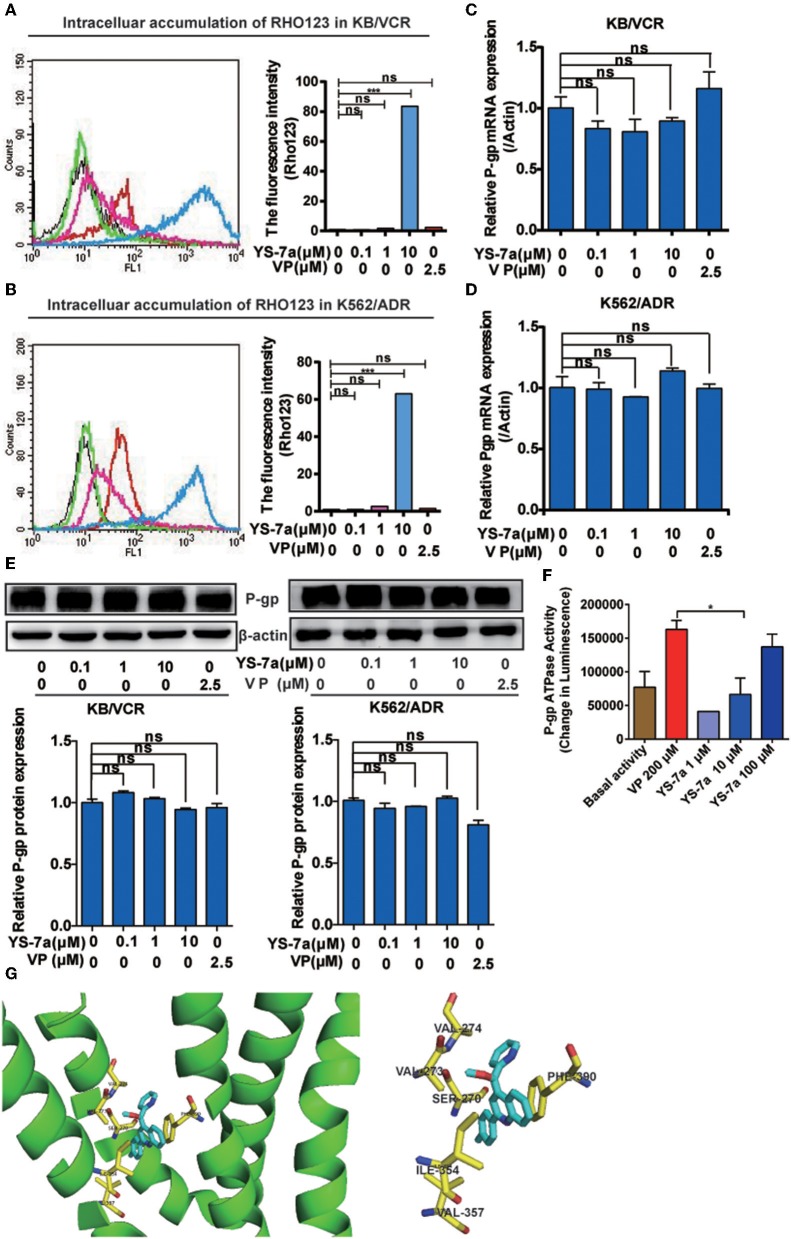
Mechanism of P-gp inhibition by YS-7a. Intracellular accumulation of Rho123 in **(A)** KB/VCR and **(B)** K562/ADR cells after exposure to YS-7a. Effect of YS-7a on the expression of P-gp **(C,D)** mRNA and **(E)** protein in KB/VCR and K562/ADR cells. **(F)** Effect of YS-7a on P-gp ATPase activity after different drug concentrations were incubated with recombinant P-gp protein; VP was used as the positive control and basal activity as the negative control. **(G)** Molecular docking of YS-7a with P-gp (3WME); the yellow amino acid residues represent a distance of 1 angstrom or less, ***P < 0.001.

MDR cells often exhibit overactivated or overexpressed P-gp and abnormal P-gp ATPase activity. To explore the specific mechanisms of YS-7a, flow cytometry was performed to monitor the function of P-gp transporters *via* Rho123 efflux. Treatment of KB/VCR cells with 10 μM YS-7a resulted in a significant increase compared to the group treated with 2.5 μM VP (positive control) (Figure 5A). Consistent results were obtained in K562/ADR cells (Figure 5B). However, YS-7a did not affect P-gp mRNA and protein levels in KB/VCR and K562/ADR cells (Figures 5C–E). These results showed that YS-7a inhibited P-gp function, but not expression. We further measured P-gp ATPase activity in the presence of YS-7a using the Pgp-Glo™ Assay Systems Kit (Promega; Madison, WI, USA), and found that YS-7a stimulated P-gp ATPase activity in a dose-dependent manner (Figure 5F). These results demonstrate that YS-7a may inhibit the P-gp substrate binding site in a similar manner as VP.

To explore the involvement of P-gp as a therapeutic target of YS-7a, we performed molecular docking experiments of YS-7a with P-gp (PDB: 3WME). The binding energies for all YS-7a poses were −11.3 kcal/mol, indicating that YS-7a could strongly bind to P-gp. Our results also showed that YS-7a formed six hydrophobic interactions with residues SER270, VAL273, VAL274, ILE354, VAL357, and PHE390 (Figure 5G). Based on these observations, YS-7a can directly bind to the functional domains of P-gp. Altogether, YS-7a may suppress the P-gp transport function without affecting its expression, by stimulating the ATPase activity of P-gp by directly binding to the six probable sites instead.

### YS-7a Has No Effect on CYP3A4 Activity and Little Toxicity Toward Normal Cells

**Figure 6:**
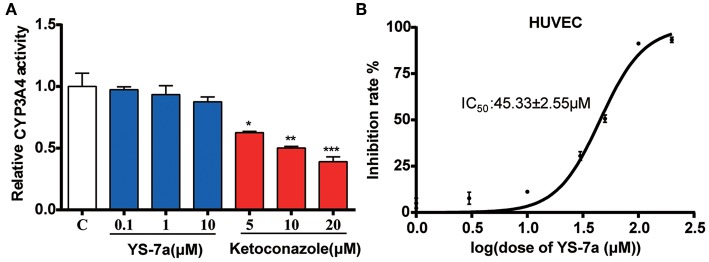
Potential toxicity of YS-7a *in vitro*. **(A)** Effect of different concentrations of YS-7a on CYP3A4 activity. **(B)** The 72-h cytotoxicity of YS-7a in HUVECs measured using the MTT assay. All experiments were repeated at least three times. *P < 0.05, **P < 0.01, and ***P < 0.001.

P-gp inhibitors, such as PSC-833 (Boesch et al., 1991; Kusunoki et al., 2010), can inhibit CYP3A4 activity, resulting in complicated drug–drug interactions and unexpected side effects; the ensuing toxicity can lead to failure of the final inhibitor clinical trial. To explore the potential for drug–drug interactions, we measured CYP3A4 activity after treatment with YS-7a. The results showed that YS-7a had no effect on CYP3A4 activity, even at high doses (100 μM) (Figure 6A). Thus, YS-7a may not have drug metabolism interactions.

Drugs can also show toxicity to vascular endothelial cells after entering the blood circulation (Cao et al., 2017). Thus, we evaluated the potential toxicity of YS-7a in human umbilical vein endothelial cells (HUVECs). The IC_50_ of YS-7a in HUVECs was 45.33 ± 2.55 μM (Figure 6B), suggesting that YS-7a (10 μM) has low potential for toxicity at concentrations used to reverse drug resistance. These results support the application of YS-7a as a novel potent P-gp inhibitor that can inhibit its transporter functions without undesirable side effects on CYP3A4 activity or endothelial cell toxicity.

### *In vivo* Drug Resistance Reversal Effect of YS-7a

**Figure 7:**
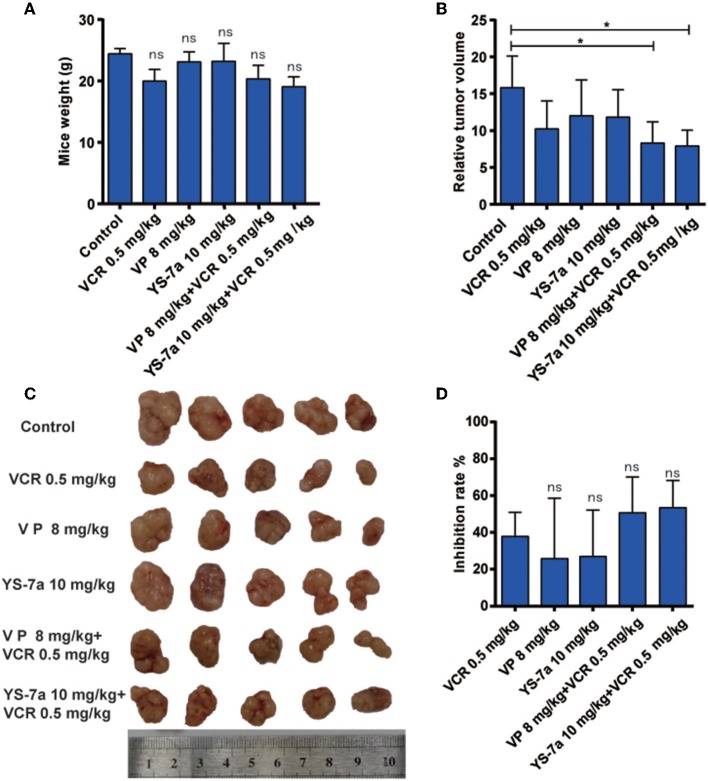
*In vivo* drug resistance reversal effect of YS-7a in KB/VCR xenograft nude mice. **(A)** The weight of KB/VCR xenograft nude mice in all groups after treatment for 24 days. **(B)** The relative tumor volume of KB/VCR xenograft nude mice in all treatment groups after 24 days. **(C)** Images of tumors from the KB/VCR xenograft nude mice in every treatment group. **(D)** Tumor inhibition rate in every treatment group after 24 days, *P < 0.05.

The mechanism and potential toxicity of YS-7a was confirmed *in vitro*. However, whether YS-7a reverses MDR *in vivo* remained unclear. Thus, we performed an *in vivo* xenograft experiment to evaluate the reversal effect of YS-7a (Figure 7). When combined with 0.5 mg/kg VCR, YS-7a at a dose of 10 mg/kg showed tumor growth inhibition of approximately 50.11%, while the single 10 mg/kg YS-7a group and single VCR group showed poor inhibition rates. Simultaneously, no YS-7a groups showed a decrease in mouse weight, indicating that YS-7a may have minimal toxicity *in vivo*. These findings indicate that YS-7a can reverse MDR *in vivo* with minimal potential toxicity.

## Discussion

To combat MDR cancer, the development of novel P-gp inhibitors is important, and most P-gp inhibitors are in preclinical or clinical trials. In this study, we synthesized 11 novel quinoline compounds, which could be divided into low-polarity (II-7a, II-7b, II-7c, II-7d, YS-7a, and YS-7b) and high-polarity (II-7a′, II-7b′, II-7c′, II-7d′, and YS-7a′) groups based on HPLC. Among these, YS-7a had the best MDR reversal effect *in vitro*, showing a reversal effect of over 10-fold in KB/VCR cells and over 30-fold in K562/ADR cells at low-toxicity concentrations.

We confirmed that YS-7a directly inhibited the transporter function of P-gp without affecting its expression, stimulating P-gp ATPase activity in a dose-dependent manner instead. Furthermore, YS-7a did not inhibit CYP3A4 activity and showed little cytotoxicity toward HUVECs at a concentration of 10 μM. In the KB/VCR xenograft model, 10 mg/kg YS-7a combined with 0.5 mg/kg VCR showed significant differences in tumor volumes compared to the control, with an average tumor growth inhibition exceeding 50%. These findings support YS-7a as a novel P-gp inhibitor, and can provide a reference for the design and development of additional P-gp inhibitors.

P-gp inhibitors are generally classified based on their inhibition mechanism: inhibiting the substrate binding site, interfering with ATP hydrolysis, or altering the integrity or fluidity of cell membrane lipids, which inhibits P-gp structural transformation (Shapiro and Ling, 1997; Varma et al., 2003; Drori et al., 2010). Most P-gp inhibitors inhibit the substrate binding site. Based on these reports, we screened P-gp inhibitors by performing Rho123 efflux experiments (Figure 3). Furthermore, MDR cancer cells show overexpression or excessive activation of P-gp, and P-gp inhibitors may inhibit the expression or function of P-gp (Silva et al., 2015). Therefore, the effects of YS-7a on P-gp function and expression were measured (Figure 5). Our results showed that YS-7a suppressed P-gp transport function without affecting its expression. In addition, P-gp ATPase activity is affected by various drug resistance regulators, such as VP (Sharom et al., 1995). The YS-7a inhibition effect of P-gp ATPase was reflected by ATP consumption measurement using the P-gp-Glo™ Assay Systems Kit (Figure 5F). Our results suggest that YS-7a inhibits the P-gp substrate binding site, similar to VP; however, further studies are required to verify its mechanism of action.

P-gp and CYP3A4 play important roles in reducing intracellular concentrations of xenobiotics and drug absorption through their respective roles in xenobiotic excretion and metabolism. P-gp and CYP3A4 work in coordination, given their co-localization in intestinal epithelial tissue and similarly overlapping substrates (Watkins, 1997; Katoh et al., 2001; Oliver et al., 2004). Several studies have reported that P-gp inhibitors inhibit CYP3A4, leading to unexpected toxicity (Wacher et al., 1998; Mathias et al., 2015). To predict potential drug–drug interactions, we measured the inhibitory effects of YS-7a on CYP3A4 activity (Figure 6). Our results suggested that YS-7a did not inhibit CYP3A4 activity. Therefore, YS-7a may have fewer side effects than PSC-833 and dexverapamil.

As shown *in vitro* (Figures 3–6), the novel P-gp inhibitor YS-7a showed superior reversal effects compared to VP by binding directly to P-gp. However, confirming whether YS-7a binds at the sites predicted by molecular docking, requires further study. Various methods, such as mutating the corresponding sites or radioisotope tracing (Hrycyna et al., 1999; Tsujimura et al., 2008), could be applied. *In vivo*, the drug resistance reversal effect of YS-7a was relatively low (about 50%). However, YS-7a at a dose of 10 mg/kg did not significantly decrease mouse body weight, suggestive of little-to-no toxicity or side effects. Thus, YS-7a may exhibit better reversal effects at higher doses. Many reports (Krepler et al., 2016; Vaidhyanathan et al., 2016) have shown that the patient-derived xenograft (PDX) model is ideal to evaluate the efficiency and toxicity of small-molecule inhibitors *in vivo*. Therefore, future studies should apply the PDX model to confirm the drug resistance reversal effect of YS-7a. Moreover, the pharmacokinetics of YS-7a should be explored to investigate its potential therapeutic mechanism in future studies.

## Conclusions

We obtained a novel potent quinoline P-gp inhibitor derived from NSC23925, which showed a cancer MDR reversal effect both *in vitro* and *in vivo*. First, 11 novel quinoline compounds were synthesized, and potential P-gp inhibitors were screened using the classic screening model. YS-7a showed a significant inhibition effect against cellular Rho123 efflux. The MDR reversal effect and potential mechanisms of YS-7a were verified *in vitro*. YS-7a suppressed the P-gp transport function without affecting its expression, by stimulating the ATPase activity of P-gp in a dose-dependent manner instead. In addition, potential binding sites were predicted based on molecular docking. Finally, *in vitro* experiments support the low toxicity of YS-7a and the MDR reversal effect of YS-7a was verified in a KB/VCR cancer xenograft model with minimal toxicity. Overall, these results suggest that YS-7a may be a potential candidate compound for the development for new agents to reverse cancer MDR.

## Data Availability Statement

The data that support the findings of this study are available from the corresponding author upon reasonable request.

## Ethics Statement

This animal study was reviewed and approved by China Pharmaceutical University.

## Author Contributions

XQ, HD, JX, XY, LSh, and LSu defined the research subject and its aims, conceived, and designed the experiments. XQ, HD, JX, XH, XG, YZ, LSh, and YW conducted the experiments. XQ, HD, JJ, LL, and SY analyzed the data and wrote the paper.

### Conflict of Interest

The authors declare that the research was conducted in the absence of any commercial or financial relationships that could be construed as a potential conflict of interest.
