# Supplementary material for: Novel Quinoline Compound Derivatives of NSC23925 as Potent Reversal Agents Against P-Glycoprotein-Mediated Multidrug Resistance
Source: Front Chem. 2019 Dec 19;7:820. doi: 10.3389/fchem.2019.00820 (PMC6931887; doi:10.3389/fchem.2019.00820)
Supplement: Supplementary file 1 [file Data_Sheet_1.pdf]

Supplementary Material

Table S1. High-performance liquid chromatography retention times and purity grade for all final products.

**Compound**	**Retention Time /min**	**Peak area/s**	**Peak height /mAU**	**Purity grade /%**
**II-7a**	19.230	5.57×10^4	815	96.61
**II-7a'**	19.718	4.20×10^4	658	95.66
**II-7b**	19.068	5.69×10^4	642	96.17
**II-7b'**	19.488	5.07×10^4	592	95.10
**II-7c**	10.917	1.67×10^5	2807	97.35
**II-7c'**	13.772	2.79×10^4	1336	95.03
**II-7d**	9.931	7784	931	95.70
**II-7d'**	12.429	1.68×10^5	1333	95.00
**YS-7a**	18.222	4.45×10^4	2762	96.33
**YS-7a'**	19.923	1.40×10^5	3800	96.93
**YS-7b**	19.409	9107	1200	97.22

COPIES OF CHROMATOGRAM OF FINAL COMPOUNDS

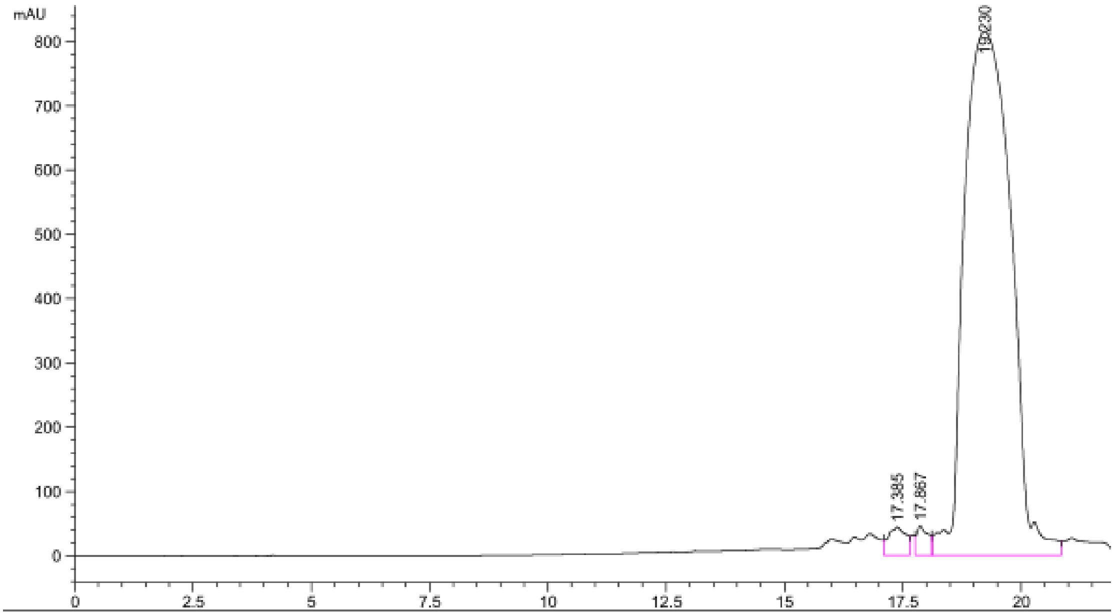

Fig S1. Chromatogram of compound **II-7a.**

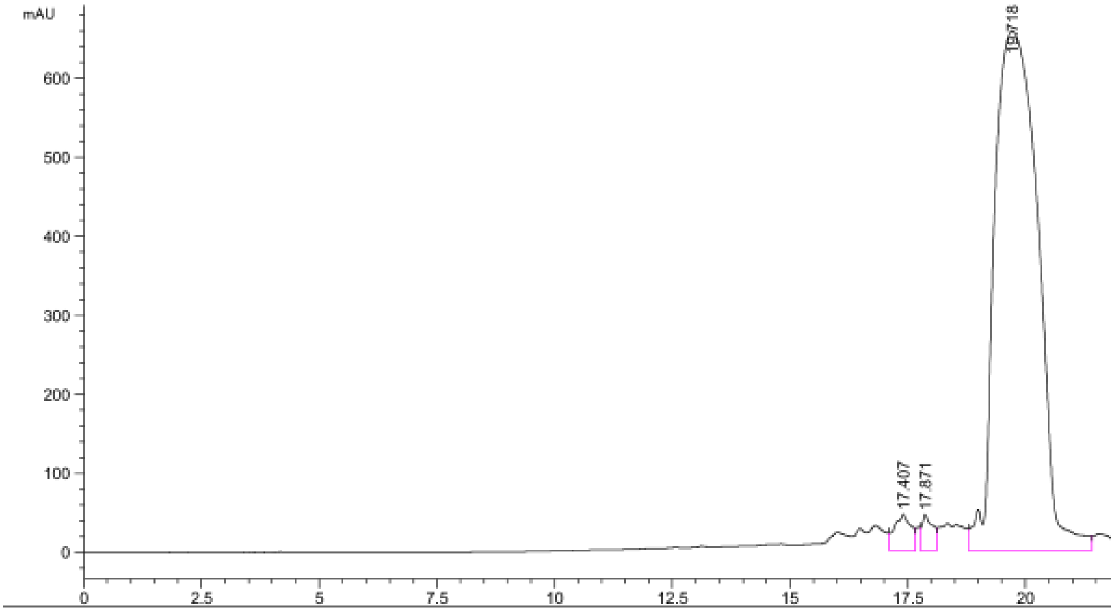

Fig S2. Chromatogram of compound **II-7a′.**

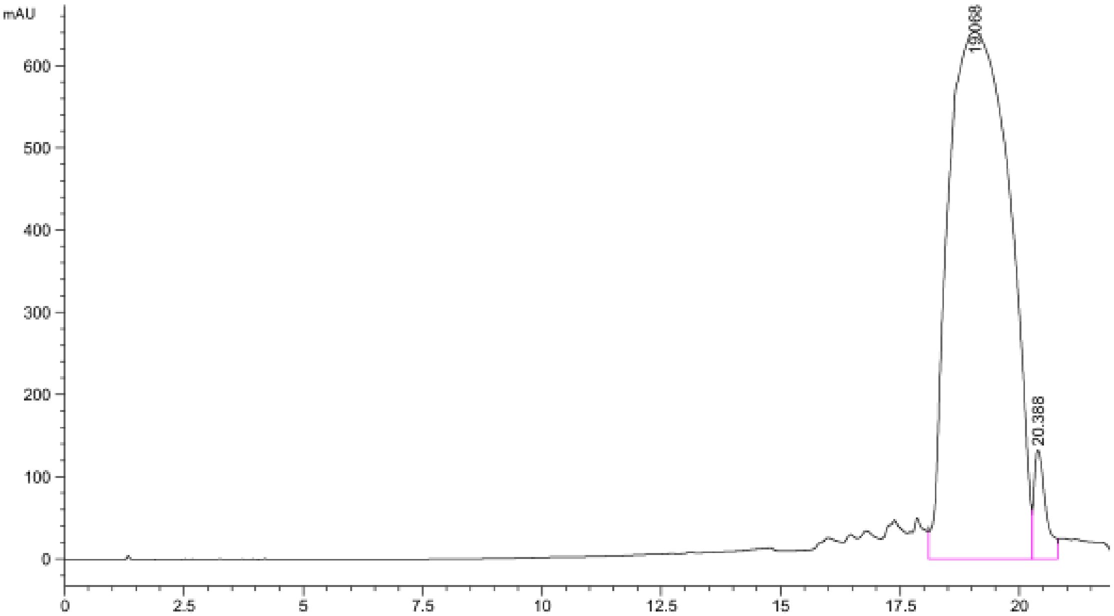

Fig S3. Chromatogram of compound **II-7b.**

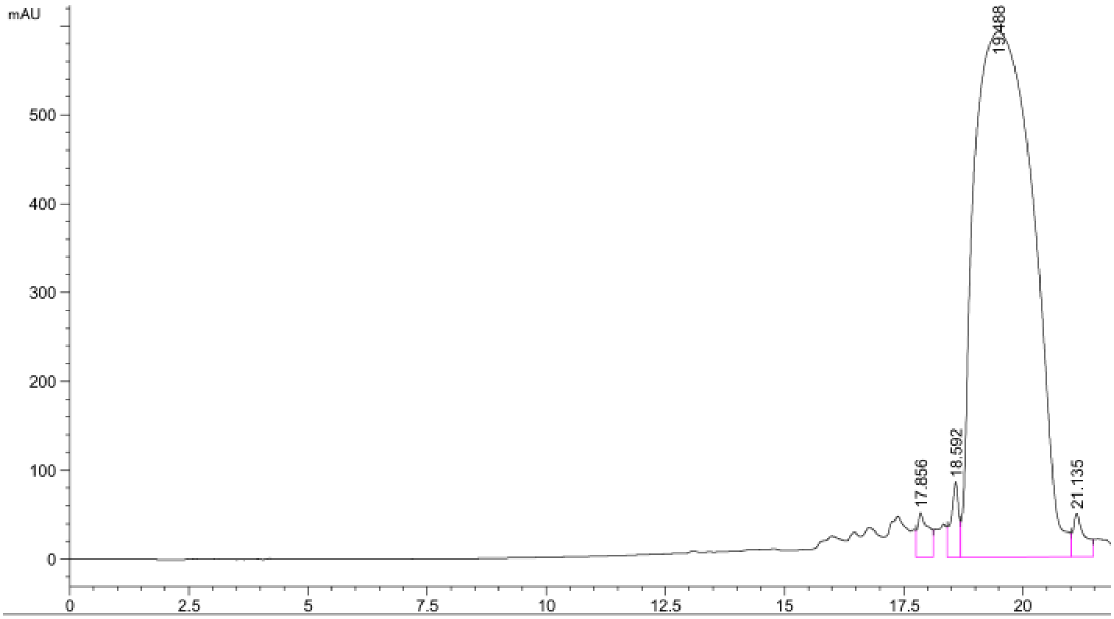

Fig S4. Chromatogram of compound **II-7b′.**

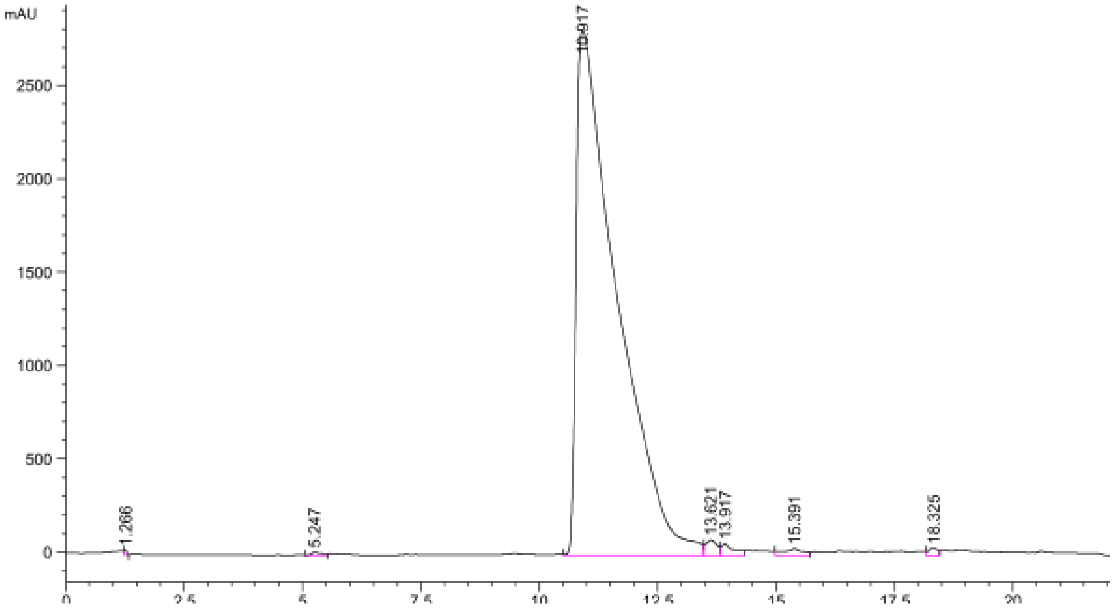

Fig S5. Chromatogram of compound **II-7c.**

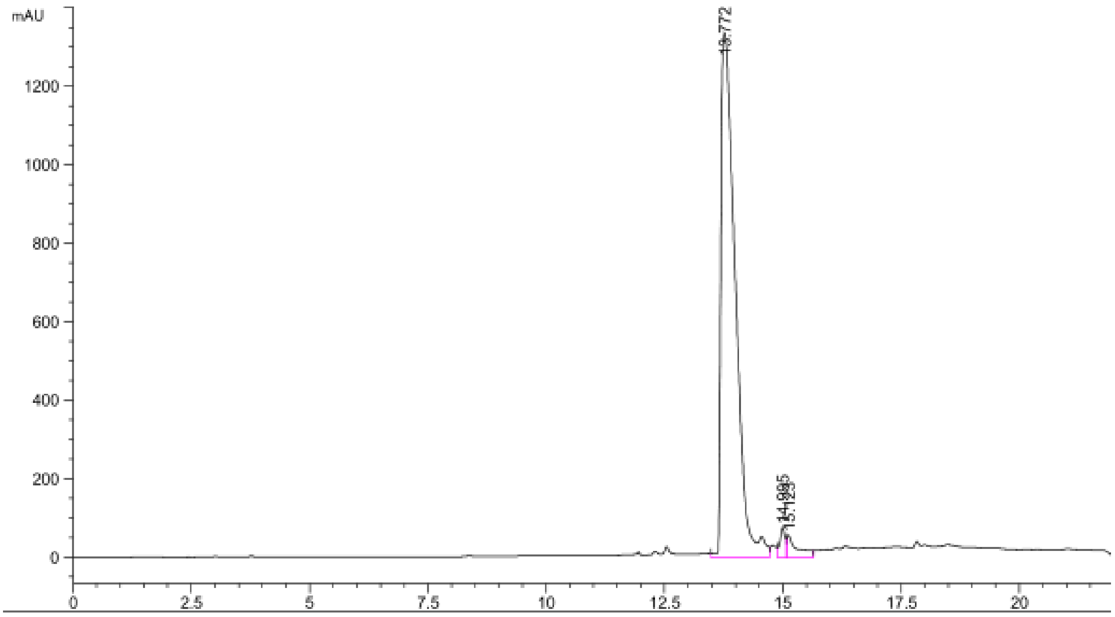

Fig S6. Chromatogram of compound **II-7c′.**

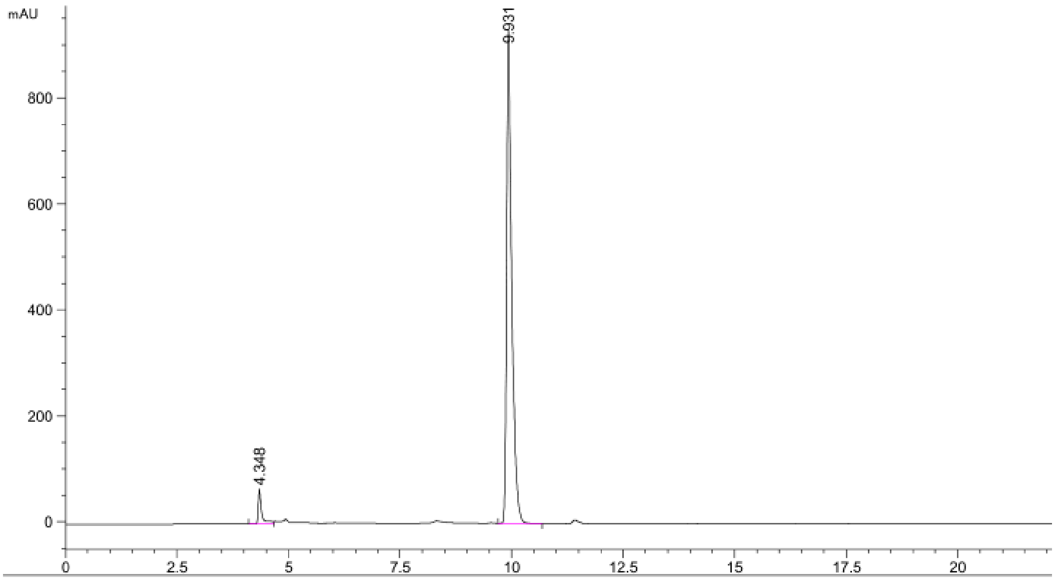

Fig S7. Chromatogram of compound **II-7d.**

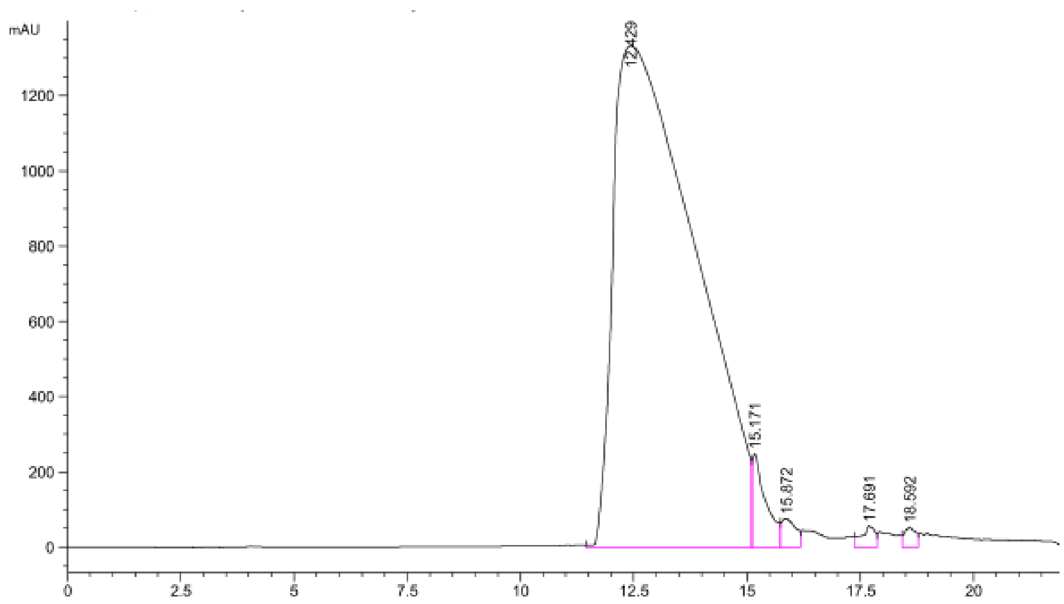

Fig S8. Chromatogram of compound **II-7d′.**

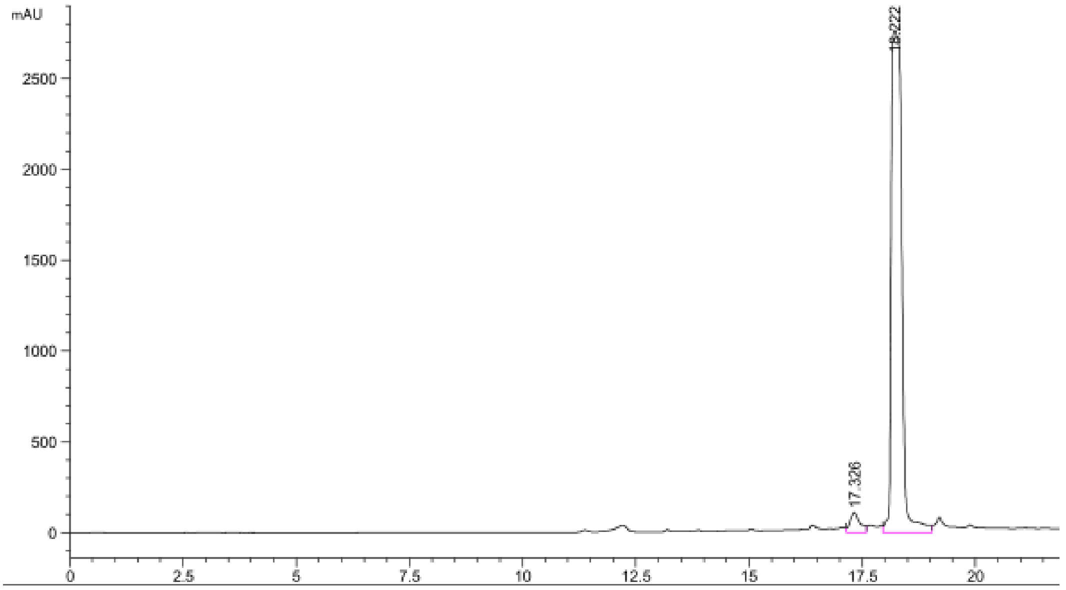

Fig S9. Chromatogram of compound **YS-7a.**

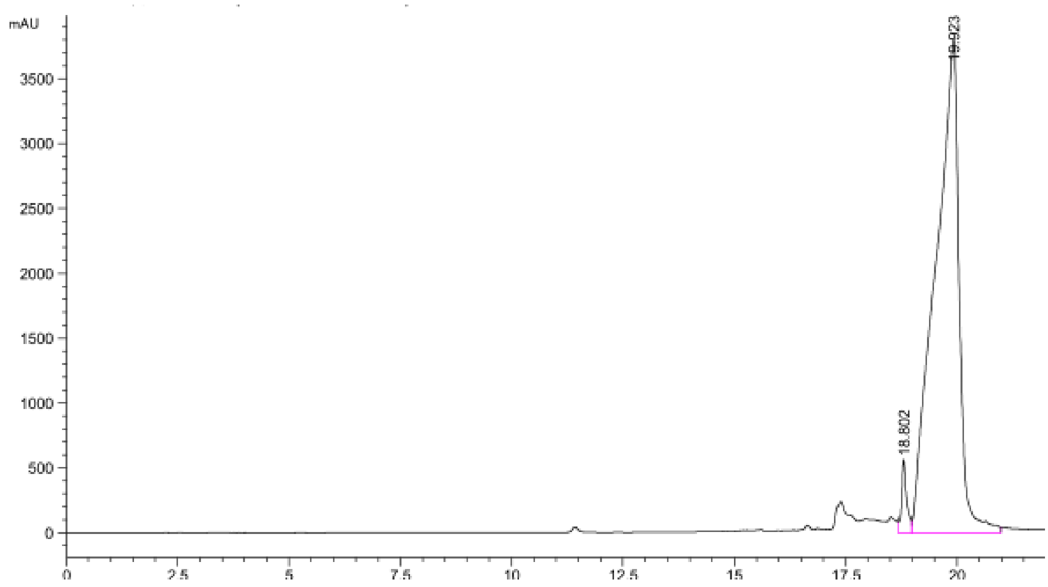

Fig S10. Chromatogram of compound **YS-7a′.**

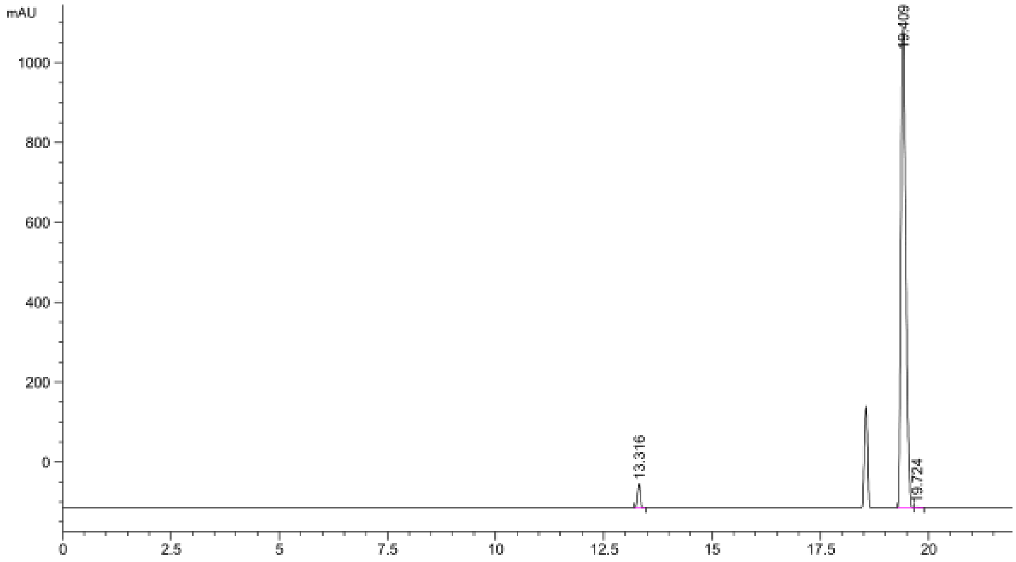

Fig S11. Chromatogram of compound **YS-7b.**

COPIES OF SPECTRA OF SELECTED COMPOUNDS

II-6a (the lower polar compound)

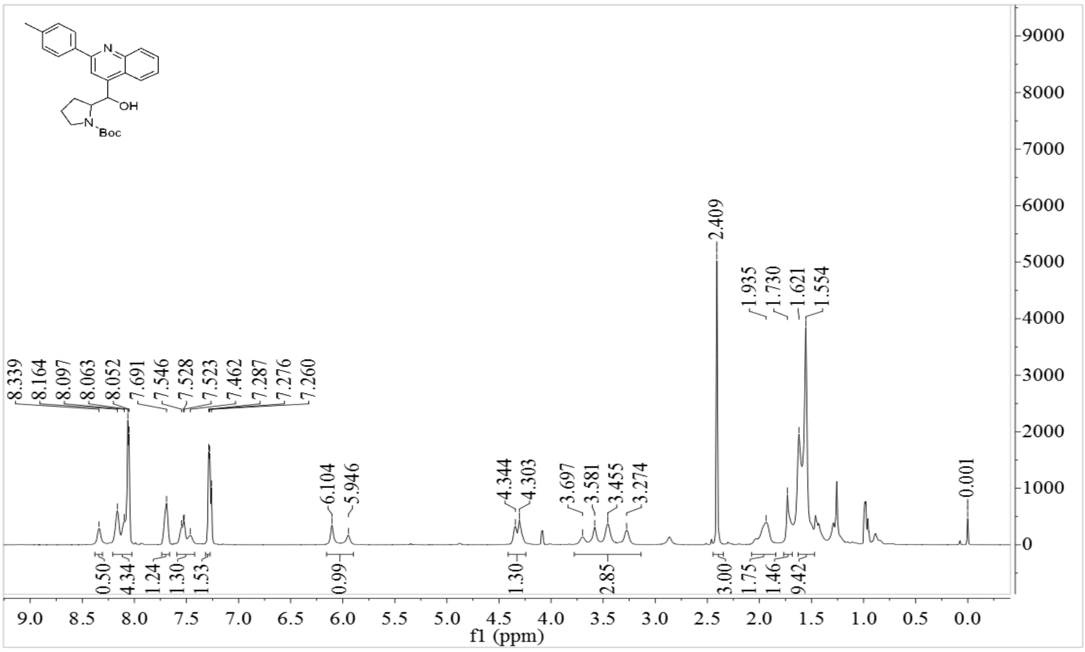

Fig S12. ^{1}H NMR spectrum of compound **II-6a.**

II-6a: 2-(Hydroxy(2-(p-tolyl)quinolin-4-yl)methyl)pyrrolidine-1-carboxylic acid tert-butyl este ^{1}H NMR (600 MHz, $CDCl_3$) δ 8.34 (s, 1H), 8.16-8.05 (m, 4H), 7.69 (s, 1H), 7.55-7.46 (m, 1H), 7.28 (d, J = 6.6 Hz, 2H), 6.02 (d, J = 97.2 Hz, 1H), 4.32 (d, J = 24.6 Hz, 1H), 3.70-3.27 (m, 3H), 2.41 (s, 3H), 1.93 (s, 2H), 1.73 (s, 1H), 1.59 (d, J = 40.2 Hz, 9H).

II-6a' (the more polar compound)

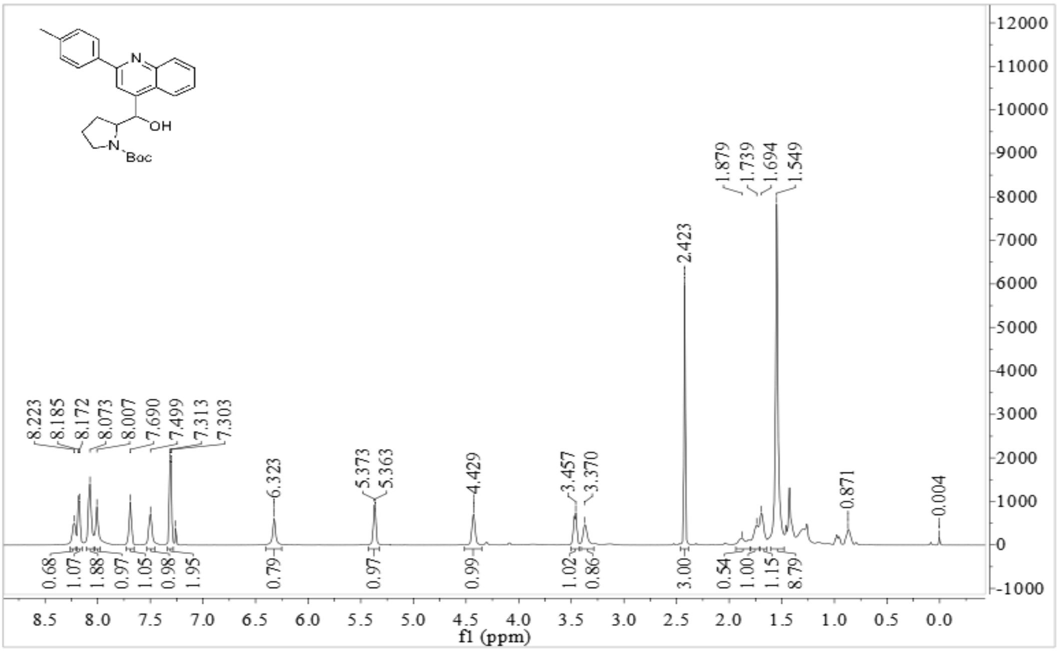

Fig S13. ^{1}H NMR spectrum of compound **II-6a′.**

II-6a′: 2-(Hydroxy(2-(p-tolyl)quinolin-4-yl)methyl)pyrrolidine-1-carboxylic acid tert-butyl ester White solid. ^{1}H NMR (600 MHz, $CDCl_3$) δ 8.22 (s, 1H), 8.18 (d, J = 7.8 Hz, 1H), 8.07 (s, 2H), 8.01

(s, 1H), 7.69 (s, 1H), 7.50 (s, 1H), 7.31 (d, *J* = 6.0 Hz, 2H), 6.32 (s, 1H), 5.37 (d, *J* = 6.0 Hz, 1H), 4.43 (s, 1H), 3.46 (s, 1H), 3.37 (s, 1H) 2.42 (s, 3H), 1.88 (s, 1H), 1.82 (s, 1H), 1.74 (s, 1H), 1.55 (s, 9H).

II-6a

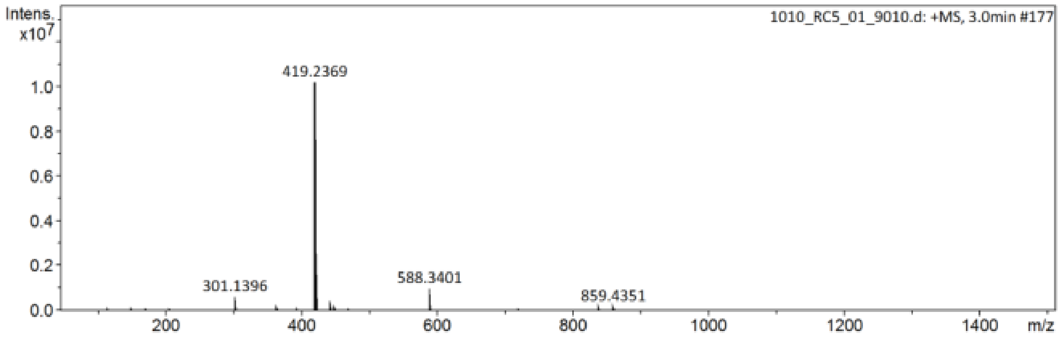

Fig S14. HNMR (EMS) spectrum of compound **II-6a**

II-6a: 2-(Hydroxy(2-(p-tolyl)quinolin-4-yl)methyl)pyrrolidine-1-carboxylic acid tert-butyl ester

HRMS (ESI), m/z calcd.For$C_{26}H_{31}N_2O_3^+$ ([M+H]+) 419.2329, found: 419.2369.

II-7a

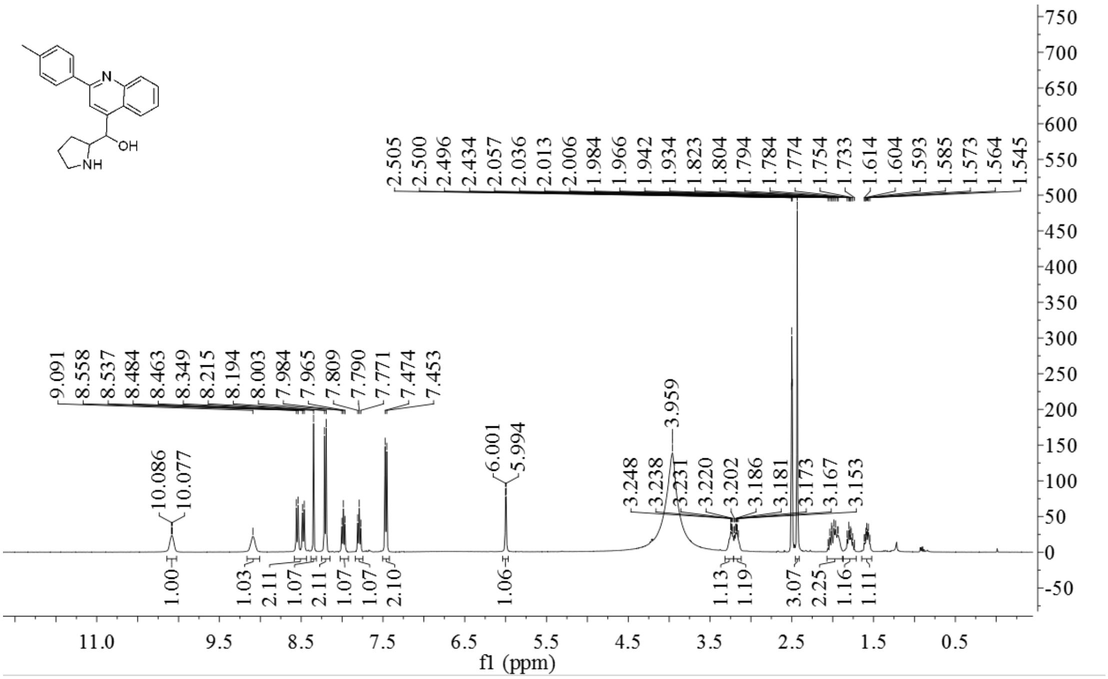

Fig S15. ^{1}H NMR spectrum of compound **II-7a.**

II-7a: (2-(4-methylphenyl)quinolin-4-yl)(pyrrolidin-2-yl)methanol

White solid; m.p., 131-133 ℃. ^{1}H NMR (400 MHz, DMSO) δ 10.08 (d, *J* = 3.6 Hz, 1H), 9.09 (s, 1H), 8.55 (d, *J* = 8.4 Hz, 1H), 8.47 (d, *J* = 8.4 Hz, 1H), 8.35 (s, 1H), 8.20 (d, *J* = 8.2 Hz, 2H), 7.98 (t, *J* = 7.6 Hz, 1H), 7.79 (t, *J* = 7.6 Hz, 1H), 7.46 (d, *J* = 8.4 Hz, 2H), 6.00 (d, *J* = 2.8 Hz, 1H), 3.23 (dd, *J* = 6.8, 4.0 Hz, 1H), 3.21 – 3.13 (m, 1H), 2.43 (s, 3H), 1.99 (ddd, *J* = 20.0, 15.2, 6.0 Hz, 2H), 1.87 – 1.70 (m, 1H), 1.58 (ddd, *J* = 16.0, 9.8, 5.8 Hz, 1H).

II-7a'

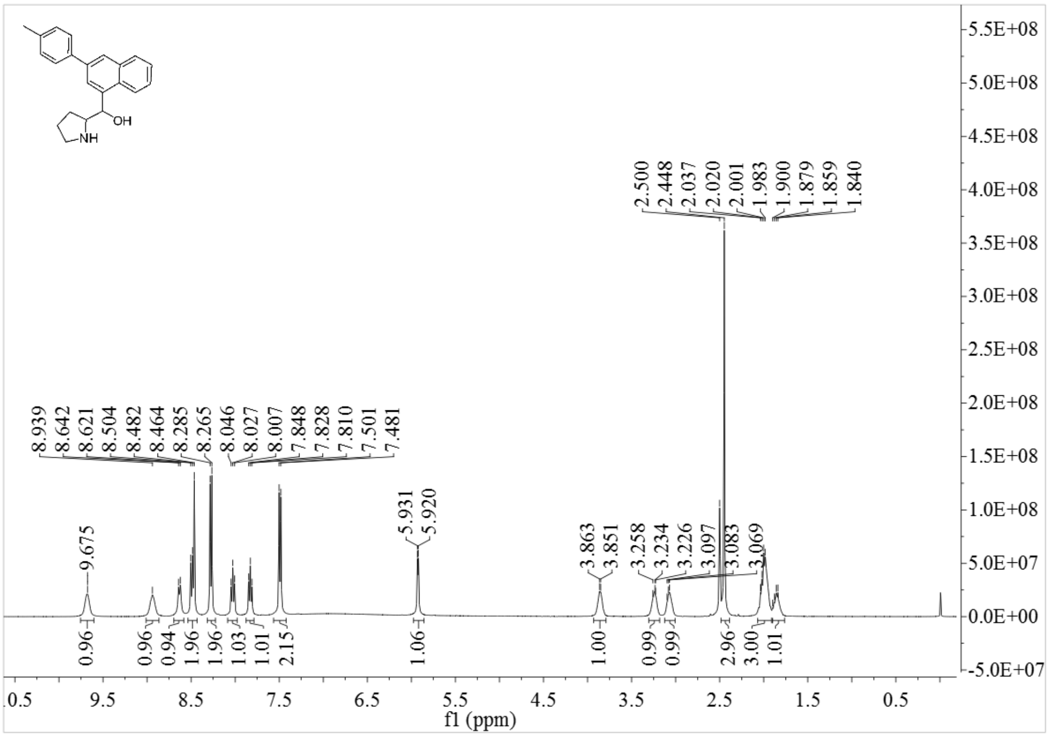

Fig S16. ^{1}H NMR spectrum of compound **II-7a'.**

II-7a: (2-(4-methylphenyl)quinolin-4-yl)(pyrrolidin-2-yl)methanol

White solid; ^{1}H NMR (400 MHz, DMSO) δ 9.67 (s, 1H), 8.94 (s, 1H), 8.63 (d, J = 8.4 Hz, 1H), 8.52 – 8.45 (m, 2H), 8.28 (d, J = 8.0 Hz, 2H), 8.03 (t, J = 7.8 Hz, 1H), 7.83 (t, J = 7.6 Hz, 1H), 7.49 (d, J = 8.0 Hz, 2H), 5.93 (d, J = 4.4 Hz, 1H), 3.86 (d, J = 4.8 Hz, 1H), 3.36 – 3.17 (m, 1H), 3.14 – 3.00 (m, 1H), 2.45 (s, 3H), 2.01 (dd, J = 14.7, 7.1 Hz, 3H), 1.87 (dd, J = 16.3, 7.9 Hz, 1H).

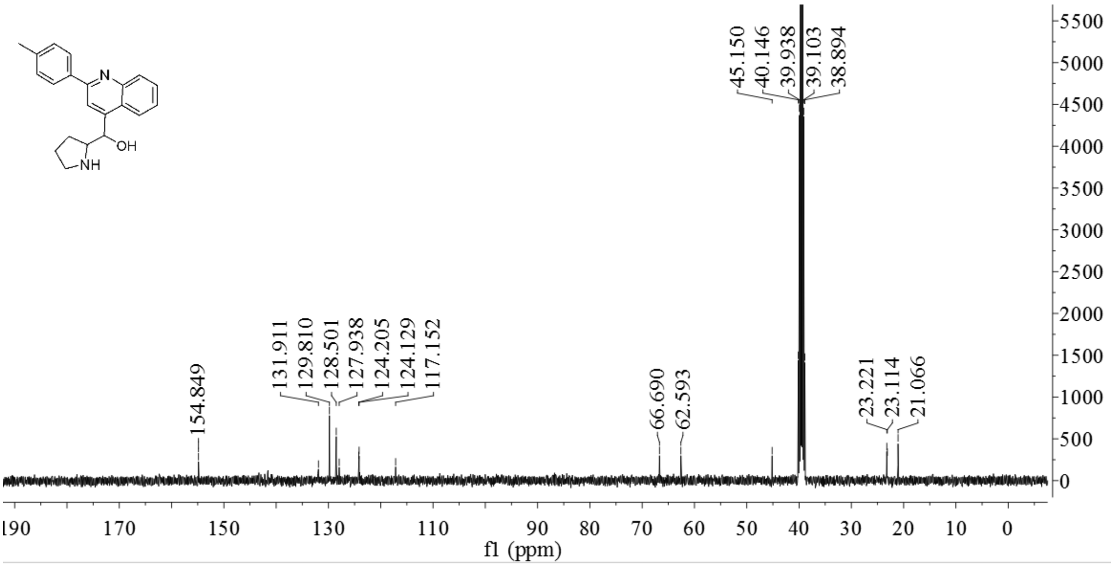

Fig S17. ^{13}C NMR spectrum of compound **II-7a.**

II-7a: (2-(4-methylphenyl)quinolin-4-yl)(pyrrolidin-2-yl)methanol

^{13}C NMR (101 MHz, DMSO) δ 154.8, 131.9, 129.8, 128.5, 127.9, 124.2, 124.1, 117.2, 66.7, 62.6, 45.2, 23.2, 23.1, 21.1.

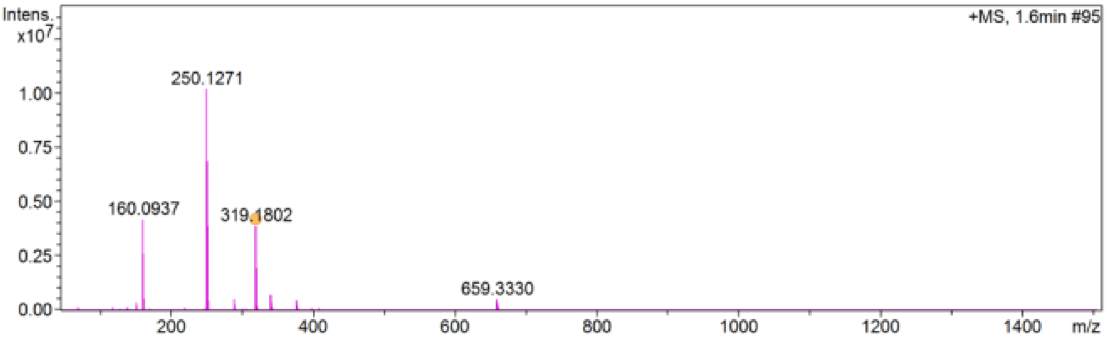

Meas. m/z	#	Ion Formula	m/z	err [ppm]	mSigma	# mSigma	Score	rdb	e¡¥ Conf	N-Rule
319.1802	1	C21H23N2O	319.1805	0.8	14.2	1	100.00	11.5	even	ok
	2	C16H23N4O3	319.1765	-11.8	32.8	2	4.42	7.5	even	ok

Fig S18. HRMS (ESI) spectrum of compound **II-7a.**

HRMS (ESI), m/z calcd. For $C_{21}H_{22}N_2NaO^+$ ([M+H]+) 319.1805, found: 319.1802.

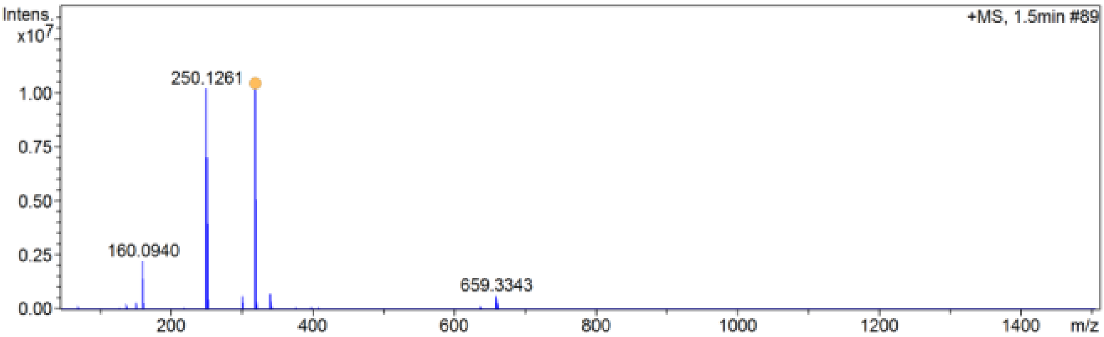

Meas. m/z	#	Ion Formula	m/z	err [ppm]	mSigma	# mSigma	Score	rdb	e¡¥ Conf	N-Rule
319.1808	1	C21H23N2O	319.1805	-0.8	44.2	1	100.00	11.5	even	ok

Fig S19. HRMS (ESI) spectrum of compound **II-7a′.**

II-7a′: (2-(4-methylphenyl)quinolin-4-yl)(pyrrolidin-2-yl)methanol

HRMS (ESI), m/z calcd. For $C_{21}H_{22}N_2NaO^+$ ([M+H]+) 319.1805, found: 319.1808.

II-6b (the lower polar compound)

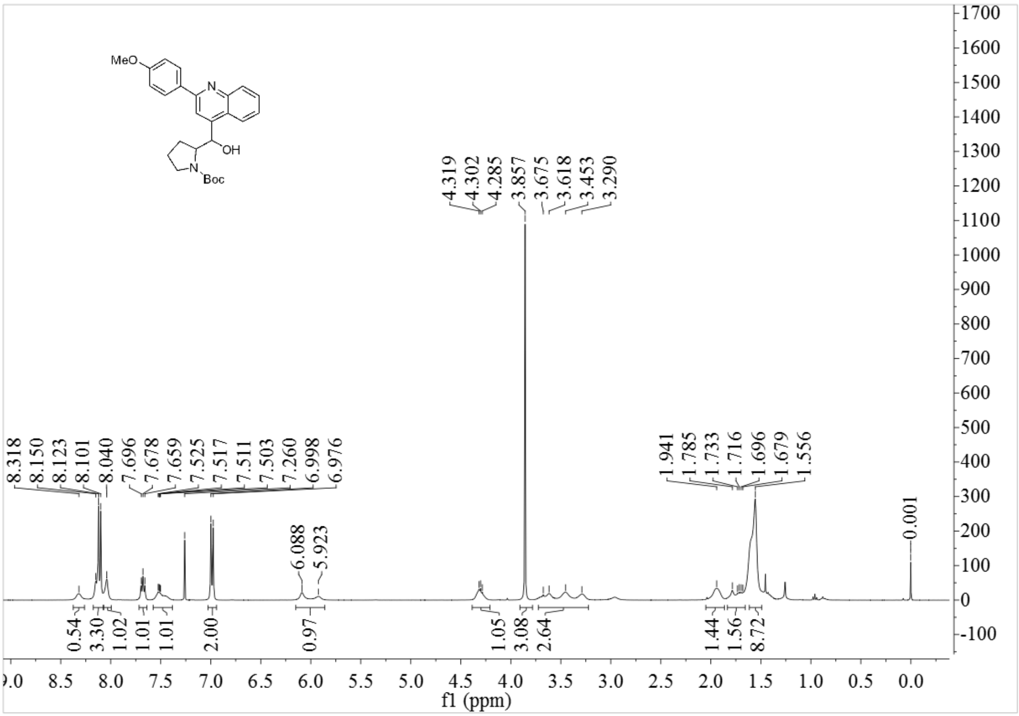

Fig S20. ^{1}H NMR spectrum of compound **II-6b.**

II-6b: 2-(Hydroxy(2-(4-methoxyphenyl)quinolin-4-yl)methyl)pyrrolidine-1-carboxylic acid tert-butyl ester

White solid. ^{1}H NMR (400 MHz, $CDCl_3$) δ 8.32 (s, 1H), 8.12 (t, J = 9.9 Hz, 3H), 8.04 (s, 1H), 7.68 (t, J = 7.3 Hz, 1H), 7.51 (dd, J = 5.7, 3.3 Hz, 1H), 6.99 (d, J = 8.8 Hz, 2H), 6.01 (d, J = 65.9 Hz, 1H), 4.39-4.22 (m, 3H), 3.86 (s, 3H), 3.62 (s, 1H), 3.45 (s, 1H), 3.29 (s, 1H), 1.94 (s, 1H), 1.84-1.66 (m, 2H), 1.56 (s, 9H).

II-6b' (the more polar compound)

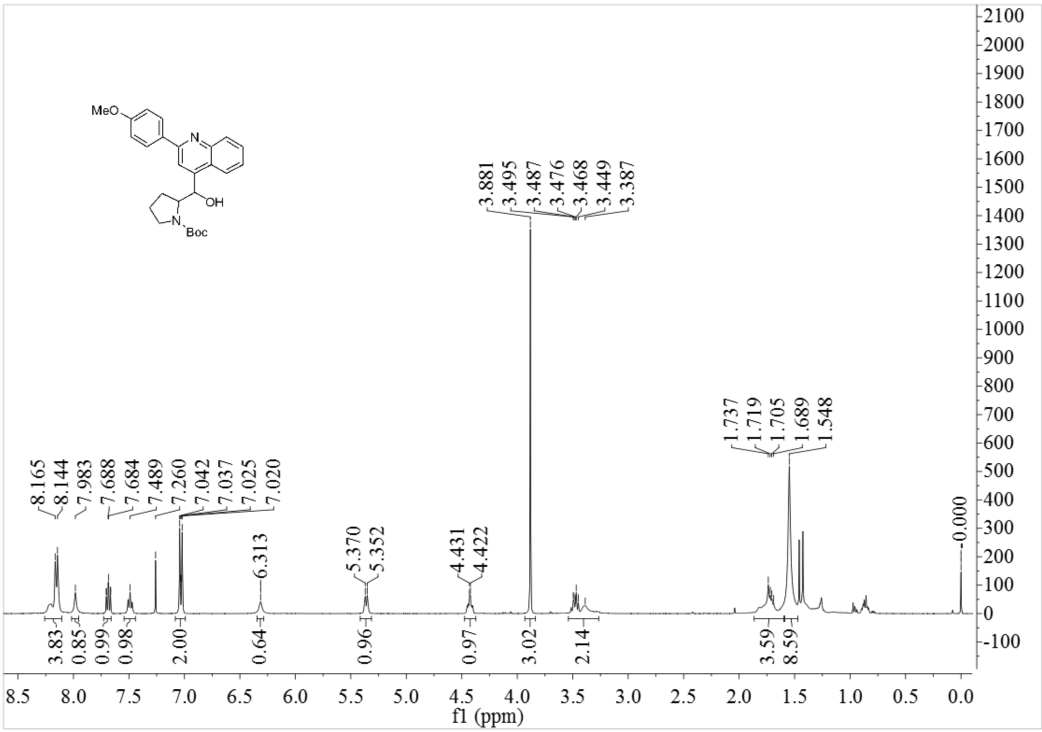

Fig S21. ^{1}H NMR spectrum of compound **II-6b′.**

II-6b': 2-(Hydroxy(2-(4-methoxyphenyl)quinolin-4-yl)methyl)pyrrolidine-1-carboxylic acid tert-butyl ester

White solid. ^{1}H NMR (400 MHz, $CDCl_3$) δ 8.16 (d, J = 8.4 Hz, 4H), 7.98 (s, 1H), 7.80 – 7.61 (m, 1H), 7.49 (s, 1H), 7.14 – 6.91 (m, 2H), 6.31 (s, 1H), 5.36 (d, J = 7.2 Hz, 1H), 4.43 (d, J = 3.6 Hz, 1H), 3.88 (s, 3H), 3.53 – 3.33 (m, 2H), 1.71 (dd, J = 12.8, 7.2 Hz, 3H), 1.55 (s, 9H).

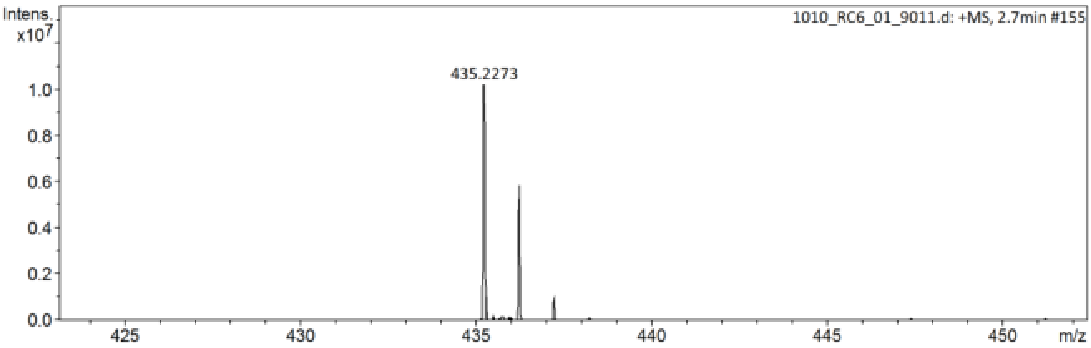

Fig S22. HNMR (EMS) spectrum of compound **II-6b.**

II-6b: 2-(Hydroxy(2-(4-methoxyphenyl)quinolin-4-yl)methyl)pyrrolidine-1-carboxylic acid tert-butyl ester

HRMS (ESI), m/z calcd.For $C_{26}H_{31}N_2O_4^+$ ([M+H]+) 435.2278, found: 435.2273.

II-7b

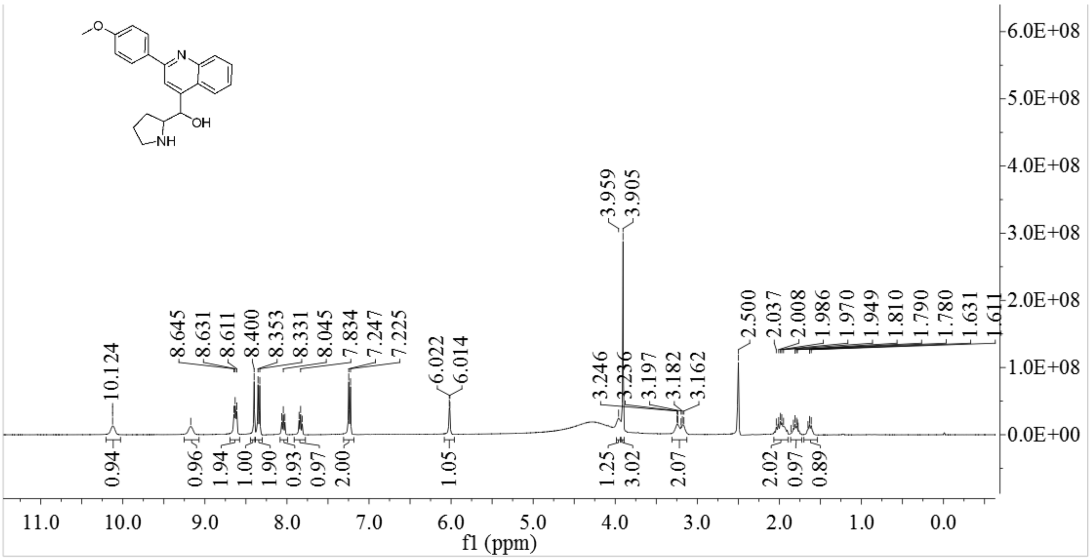

Fig S23. ^{1}H NMR spectrum of compound **II-7b.**

II-7b: (2-(4-Methoxyphenyl)quinolin-4-yl)(pyrrolidin-2-yl)methanol

White solid, m.p., 135-137 °C. ^{1}H NMR (400 MHz, DMSO) δ 10.12 (s, 1H), 9.17 (s, 1H), 8.77 – 8.56 (m, 2H), 8.40 (s, 1H), 8.34 (d, J = 8.8 Hz, 2H), 8.04 (t, J = 7.6 Hz, 1H), 7.83 (t, J = 7.6 Hz, 1H), 7.24 (d, J = 8.8 Hz, 2H), 6.02 (d, J = 3.2 Hz, 1H), 3.96 (s, 1H), 3.91 (s, 3H), 3.31 – 3.10 (m, 2H), 2.17 – 1.88 (m, 2H), 1.88 – 1.73 (m, 1H), 1.70 – 1.52 (m, 1H).

II-7b'

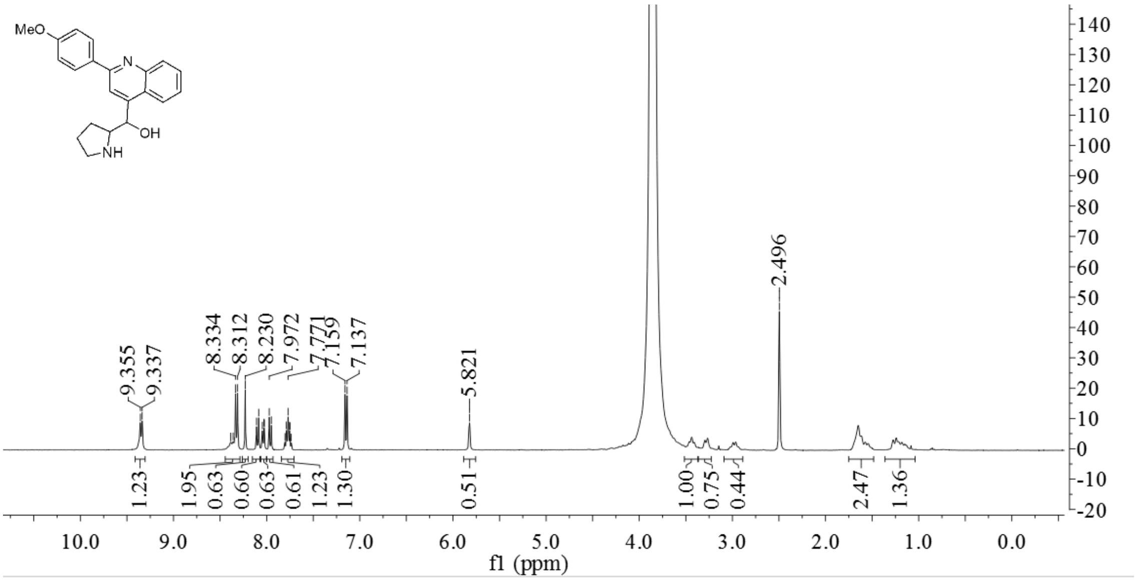

Fig S24. ^{1}H NMR spectrum of compound **II-7b′.**

II-7b′: (2-(4-Methoxyphenyl)quinolin-4-yl)(pyrrolidin-2-yl)methanol

White solid, m.p., 135-137 °C.^{1}H NMR (400 MHz, DMSO) δ 9.35 (d, J = 7.4 Hz, 1H), 8.35 (dd, J = 20.2, 9.8 Hz, 2H), 8.23 (s, 1H), 8.10 (d, J = 9.2 Hz, 1H), 8.06-8.00 (m, ^{1}H), 7.96 (d, J = 9.2 Hz, ^{1}H), 7.87-7.71 (m, 1H), 7.15 (d, J = 8.8 Hz, 1H), 5.82 (s, 1H), 3.44 (t, J = 10.2 Hz, 1H), 3.28 (d, J = 11.2 Hz, 1H), 2.95 (t, J = 22.2 Hz, 1H), 1.81-1.46 (m, 2H), 1.38-1.06 (m, 1H).

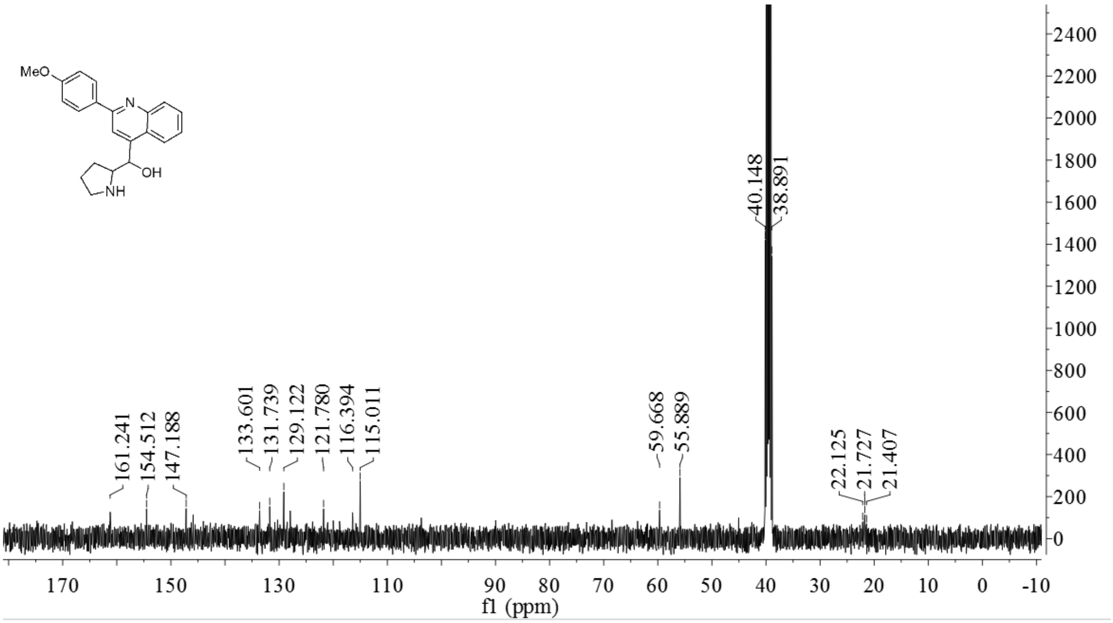

Fig S25. ^{13}C NMR spectrum of compound **II-7b.**

II-7b: (2-(4-Methoxyphenyl)quinolin-4-yl)(pyrrolidin-2-yl)methanol

^{13}C NMR (101 MHz, DMSO) δ 161.2, 154.5, 147.2, 133.6, 131.7, 129.1, 121.8, 115.0, 59.7, 55.9, 22.1, 21.7, 21.4.

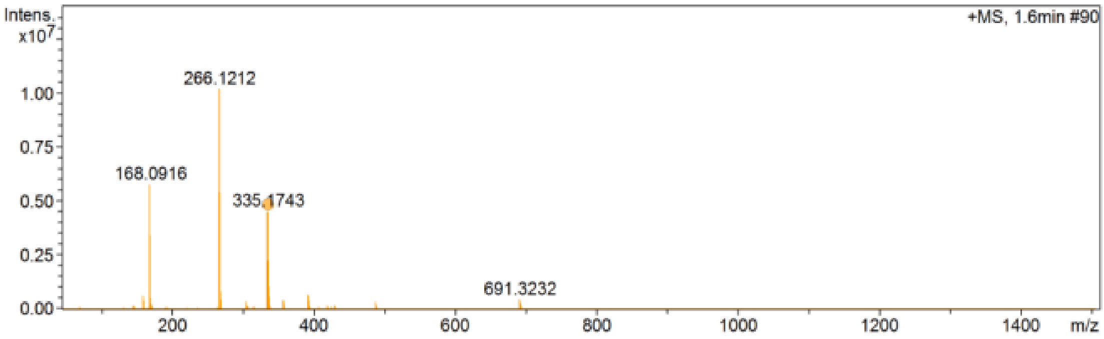

Meas. m/z	#	Ion Formula	m/z	err [ppm]	mSigma	# mSigma	Score	rdb	e¡¥ Conf	N-Rule
335.1743	1	C21H23N2O2	335.1754	3.4	3.4	1	100.00	11.5	even	ok

Fig S26. HRMS (ESI) spectrum of compound **II-7b.**

II-7b: (2-(4-Methoxyphenyl)quinolin-4-yl)(pyrrolidin-2-yl)methanol

HRMS (ESI), m/z calcd. For $C_{21}H_{23}N_2O_2^+$ ([M+H]+) 335.1754, found: 335.1743.

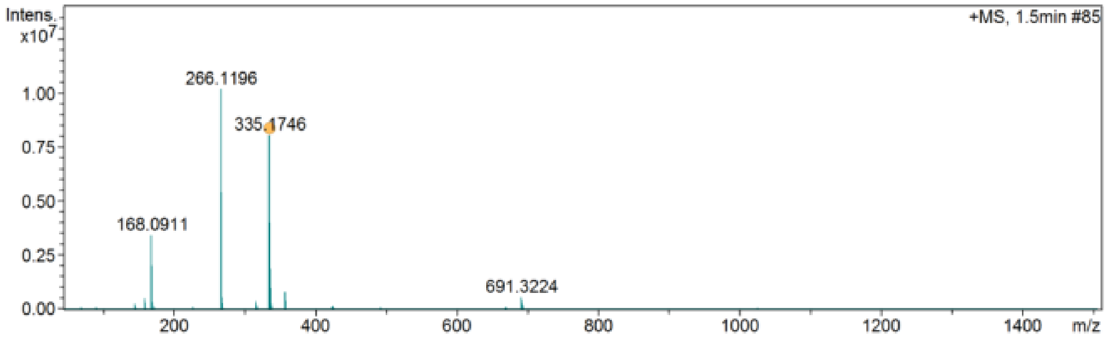

Meas. m/z	#	Ion Formula	m/z	err [ppm]	mSigma	# mSigma	Score	rdb	e¡¥ Conf	N-Rule
335.1746	1	C21H23N2O2	335.1754	2.4	1.2	1	100.00	11.5	even	ok

Fig S27. HRMS (ESI) spectrum of compound **II-7b′.**

II-7b′: (2-(4-Methoxyphenyl)quinolin-4-yl)(pyrrolidin-2-yl)methanol

HRMS (ESI), m/z calcd. For $C_{21}H_{23}N_2O_2^+$ ([M+H]+) 335.1754, found: 335.1746.

II-6c (the small polar compound)

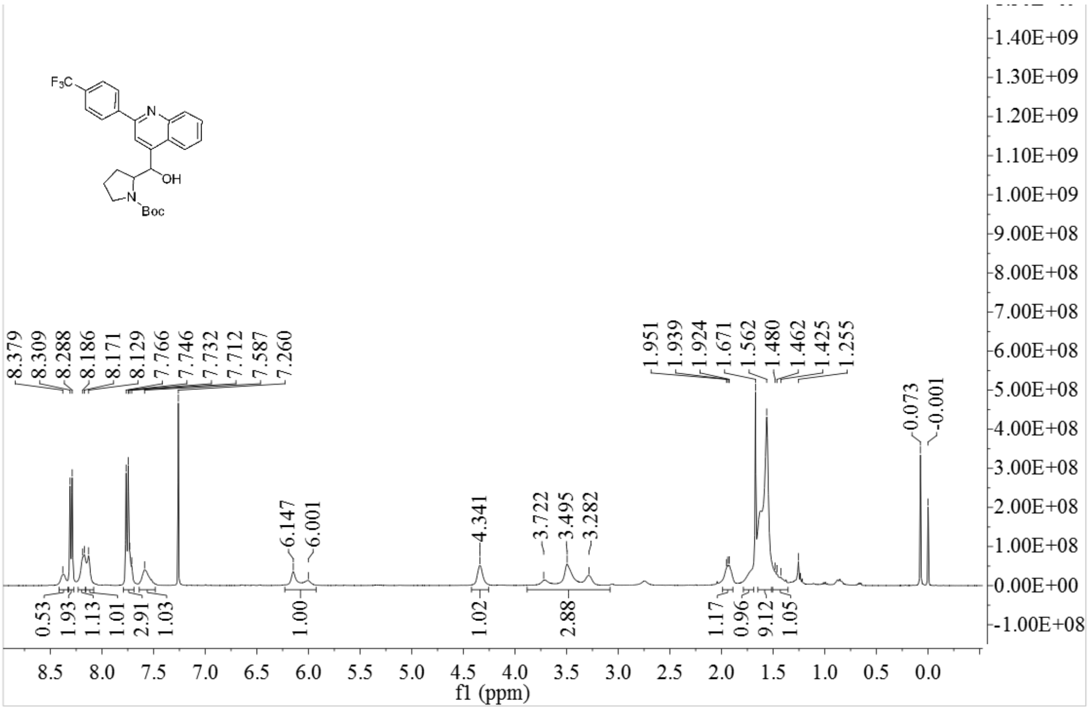

Fig S28. ^{1}H NMR spectrum of compound **II-6c.**

II-6c: 2-(Hydroxy(2-(4-trifluoromethyl)quinolin-4-yl)methyl)pyrrolidine-1-carboxylic acid tert-butyl ester

White powder; ^{1}H NMR (400 MHz, $CDCl_3$) δ 8.38 (s, 1H), 8.30 (d, J = 8.0 Hz, 2H), 8.18 (d, J = 6.3 Hz, 1H), 8.13 (s, 1H), 7.74 (dd, J = 13.2, 8.0 Hz, 3H), 7.59 (s, 1H), 6.07 (d, J = 58.3 Hz, 1H), 4.34 (s, 1H), 3.72-3.28 (m, 3H), 2.05 – 1.87 (m, 1H), 1.67 (s, 1H), 1.56 (s, 9H), 1.54 – 1.35 (m, 1H).

II-6c' (the more polar compound)

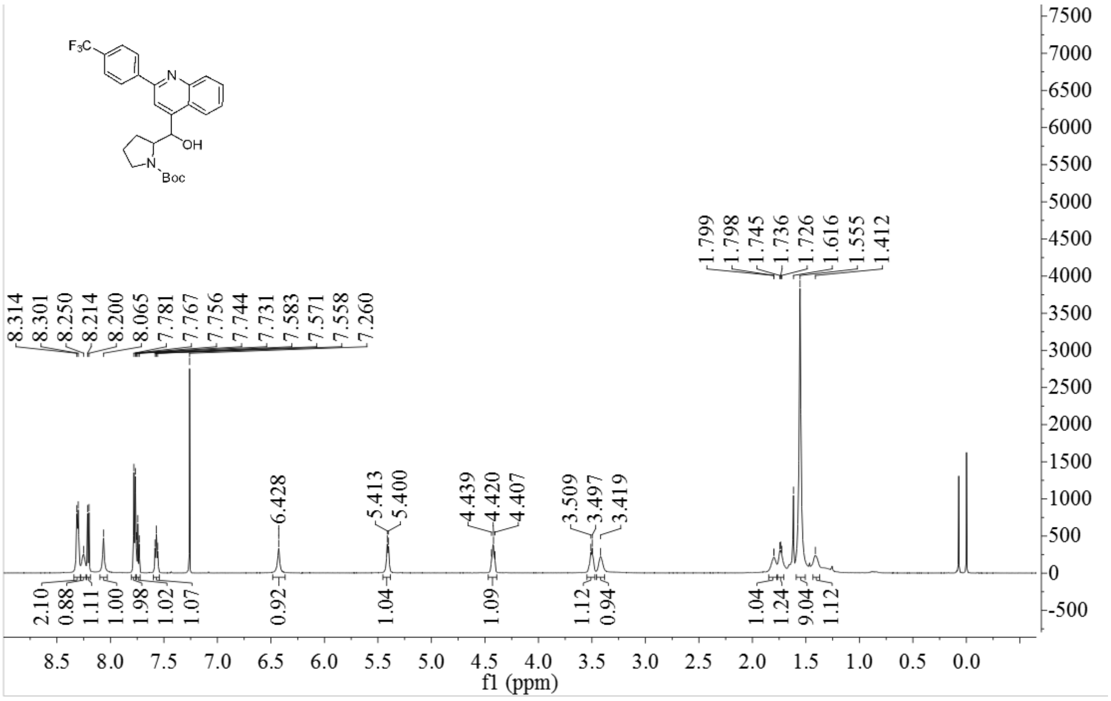

Fig S29. ^{1}H NMR spectrum of compound **II-6c′.**

II-6c′: 2-(Hydroxy(2-(4-trifluoromethyl)quinolin-4-yl)methyl)pyrrolidine-1-carboxylic acid tert-butyl ester

White powder; ^{1}H NMR (600 MHz, $CDCl_3$) δ 8.31 (d, J = 7.8 Hz, 2H), 8.25 (s, 1H), 8.21 (d, J = 8.4 Hz, 1H), 8.07 (s, 1H), 7.77 (d, J = 8.4 Hz, 2H), 7.74 (t, J = 7.8 Hz, 1H), 7.57 (t, J = 7.8 Hz, 1H), 6.43 (s, 1H), 5.41 (d, J = 7.8 Hz, 1H), 4.42 (t, J = 9.6 Hz, 1H), 3.50 (d, J = 7.2 Hz, 1H), 3.42 (s, 1H), 1.80 (s, 1H), 1.77 – 1.71 (m, 1H), 1.55 (s, 9H), 1.41 (s, 1H).

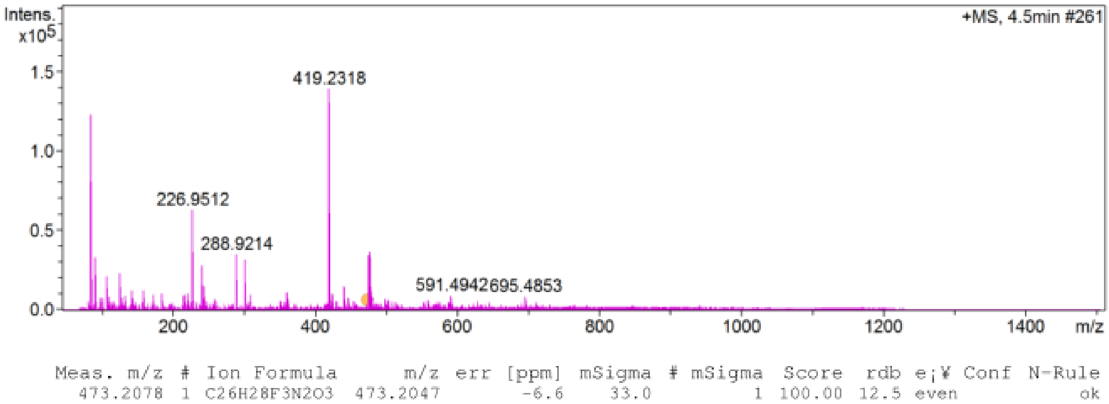

Fig S30. HRMS (ESI) spectrum of compound **II-6c.**

II-6c: 2-(Hydroxy(2-(4-trifluoromethyl)quinolin-4-yl)methyl)pyrrolidine-1-carboxylic acid tert-butyl ester

HRMS (ESI), m/z calcd. For $C_{26}H_{28}F_3N_2O_3^+$ ([M+H]+) 473.2047, found: 473.2078.

II-7c

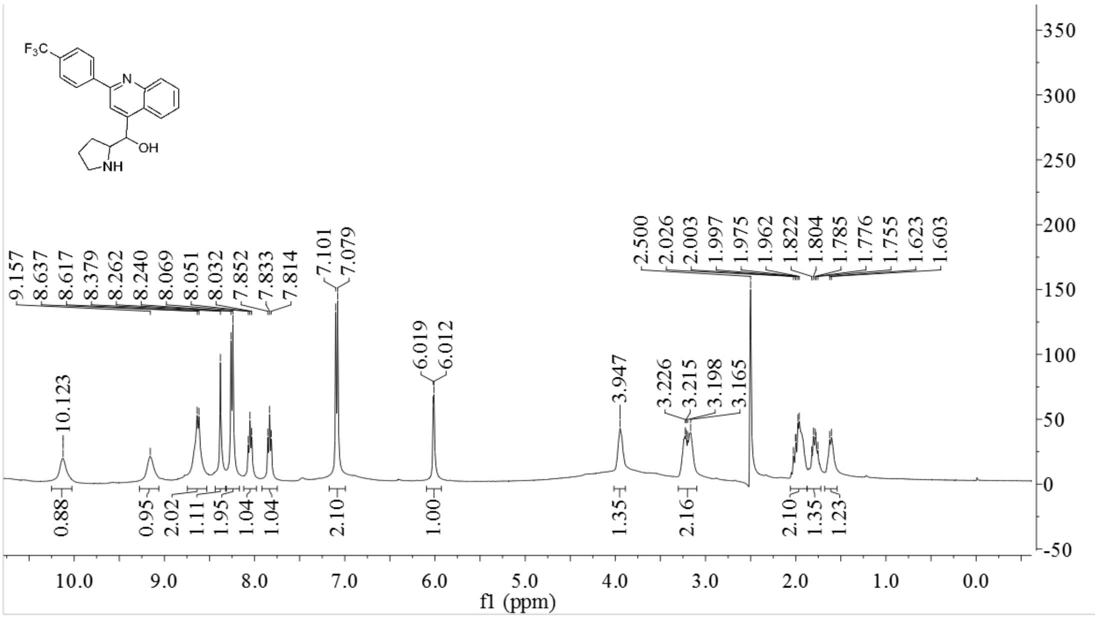

Fig S31. ^{1}H NMR spectrum of compound **II-7c.**

II-7c: (2-(4-trifluoromethyl)quinolin-4-yl)(pyrrolidin-2-yl)methanol

White powder; m.p. 137-139 °C; ^{1}H NMR (400 MHz, DMSO) δ 10.12 (s, 1H), 9.16 (s, 1H), 8.63 (d, J = 8.2 Hz, 2H), 8.38 (s, 1H), 8.25 (d, J = 8.8 Hz, 2H), 8.17 – 7.97 (m, 1H), 7.83 (t, J = 7.6 Hz, 1H), 7.09 (d, J = 8.6 Hz, 3H), 6.02 (d, J = 2.8 Hz, 1H), 3.95 (s, 2H), 3.20 (dd, J = 15.6, 8.6 Hz, 3H), 2.06 – 1.85 (m, 3H), 1.84 – 1.69 (m, 2H), 1.61 (d, J = 8.0 Hz, 2H).

II-7c′

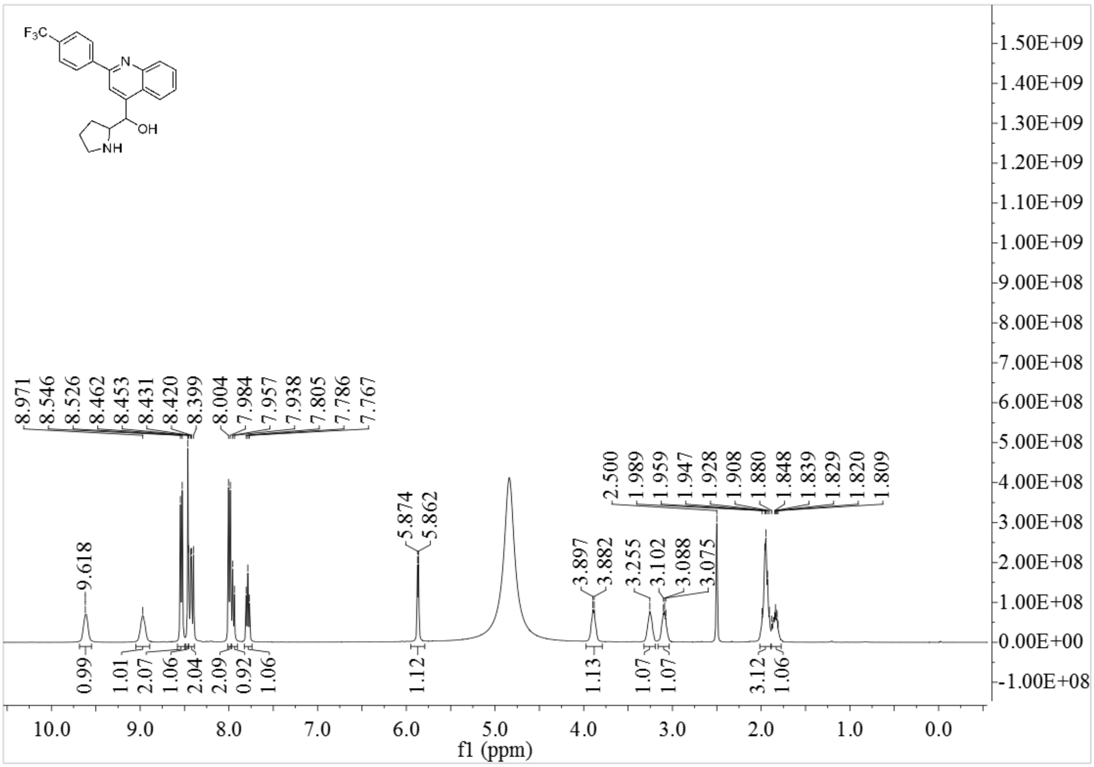

Fig S32. ^{1}H NMR spectrum of compound **II-7c′.**

II-7c': (2-(4-trifluoromethyl)quinolin-4-yl)(pyrrolidin-2-yl)methanol

White powder; m.p. 137-139 °C; ^{1}H NMR (400 MHz, DMSO) δ 9.62 (s, 1H), 8.97 (s, 1H), 8.54 (d, J = 8.0 Hz, 2H), 8.46 (s, 1H), 8.43 (dd, J = 13.2, 8.8 Hz, 2H), 7.99 (d, J = 8.4 Hz, 2H), 7.95 (d, J = 7.8 Hz, 1H), 7.79 (t, J = 7.6 Hz, 1H), 5.87 (d, J = 4.8 Hz, 1H), 3.89 (d, J = 6.0 Hz, 1H), 3.26 (s, 1H), 3.21 – 2.99 (m, 1H), 2.03 – 1.91 (m, 3H), 1.91 – 1.77 (m, 1H).

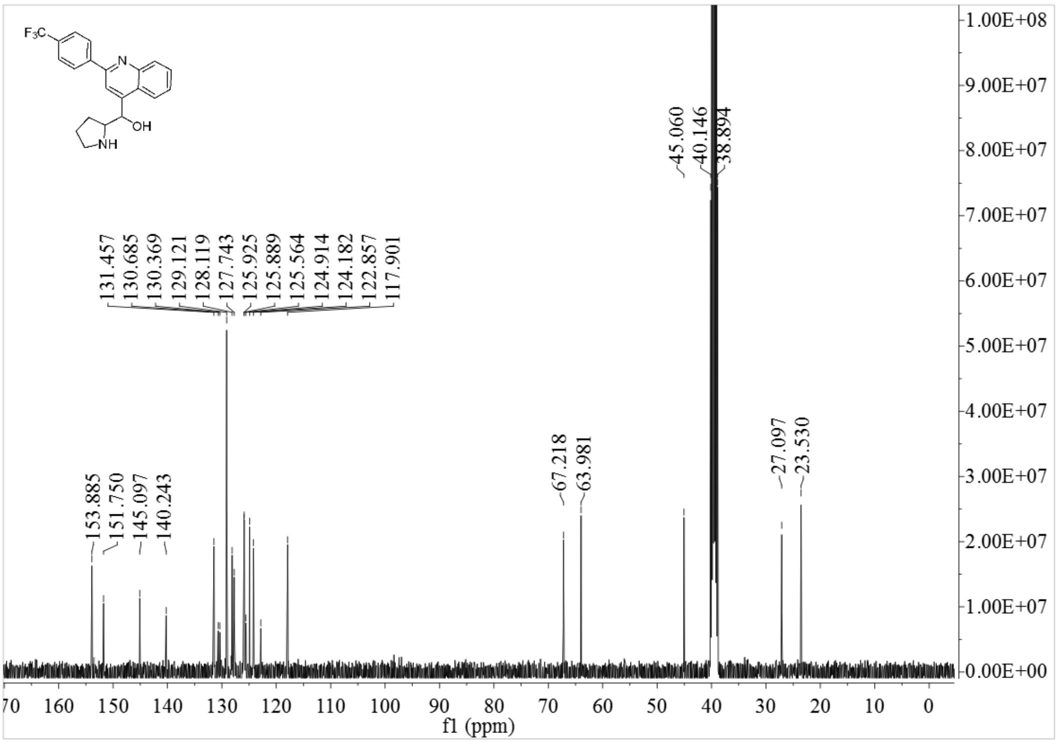

Fig S33. ^{13}C NMR spectrum of compound **II-7c.**

II-7c: (2-(4-trifluoromethyl)quinolin-4-yl)(pyrrolidin-2-yl)methanol

^{13}C NMR (101 MHz, DMSO) δ 153.9, 151.8, 145.1, 140.2, 131.5, 130.7, 130.4, 129.1, 128.1, 127.7, 125.91 (d, J = 3.6 Hz), 125.6, 124.9, 124.2, 122.9, 117.9, 67.2, 64.0, 45.1, 27.1, 23.5.

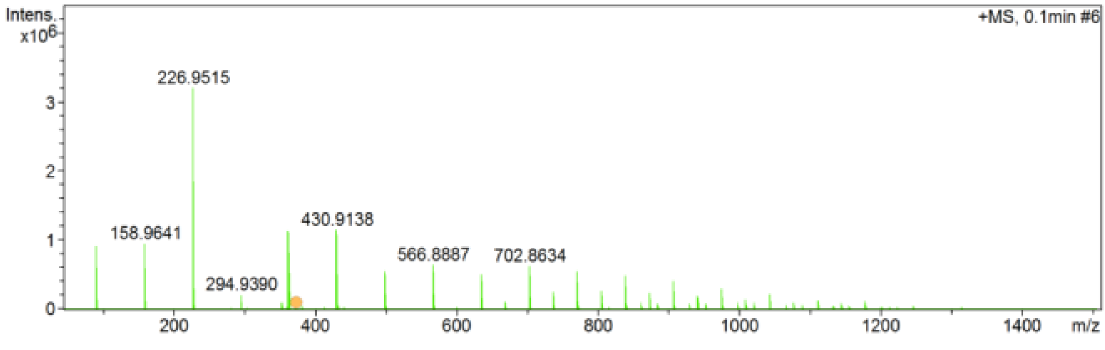

Meas. m/z	#	Ion Formula	m/z	err [ppm]	mSigma	# mSigma	Score	rdb	e¡¥ Conf	N-Rule
373.1524	1	C21H20F3N2O	373.1522	-0.6	n.a.	1	100.00	11.5	even	ok

Fig S34. HRMS (ESI) spectrum of compound **II-7c.**

II-7c: (2-(4-trifluoromethyl)quinolin-4-yl)(pyrrolidin-2-yl)methanol

HRMS (ESI), m/z calcd. For $C_{21}H_{20}F_3N_2O^+$ ([M+H]+) 373.1522, found: 373.1524.

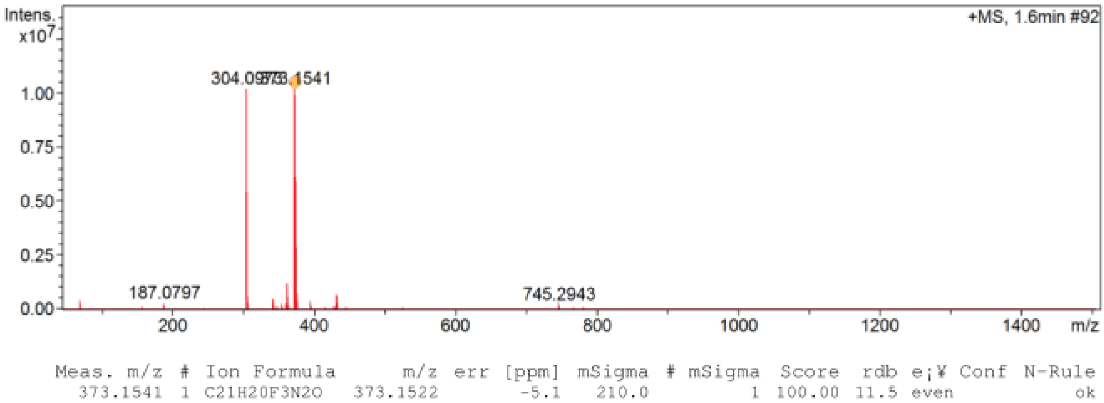

Fig S35. HRMS (ESI) spectrum of compound **II-7c.**

II-7c: (2-(4-trifluoromethyl)quinolin-4-yl)(pyrrolidin-2-yl)methanol

HRMS (ESI), m/z calcd. For $C_{21}H_{20}F_3N_2O^+$ ([M+H]+) 373.1522, found: 373.1541.

II-6d (the lower polar compound)

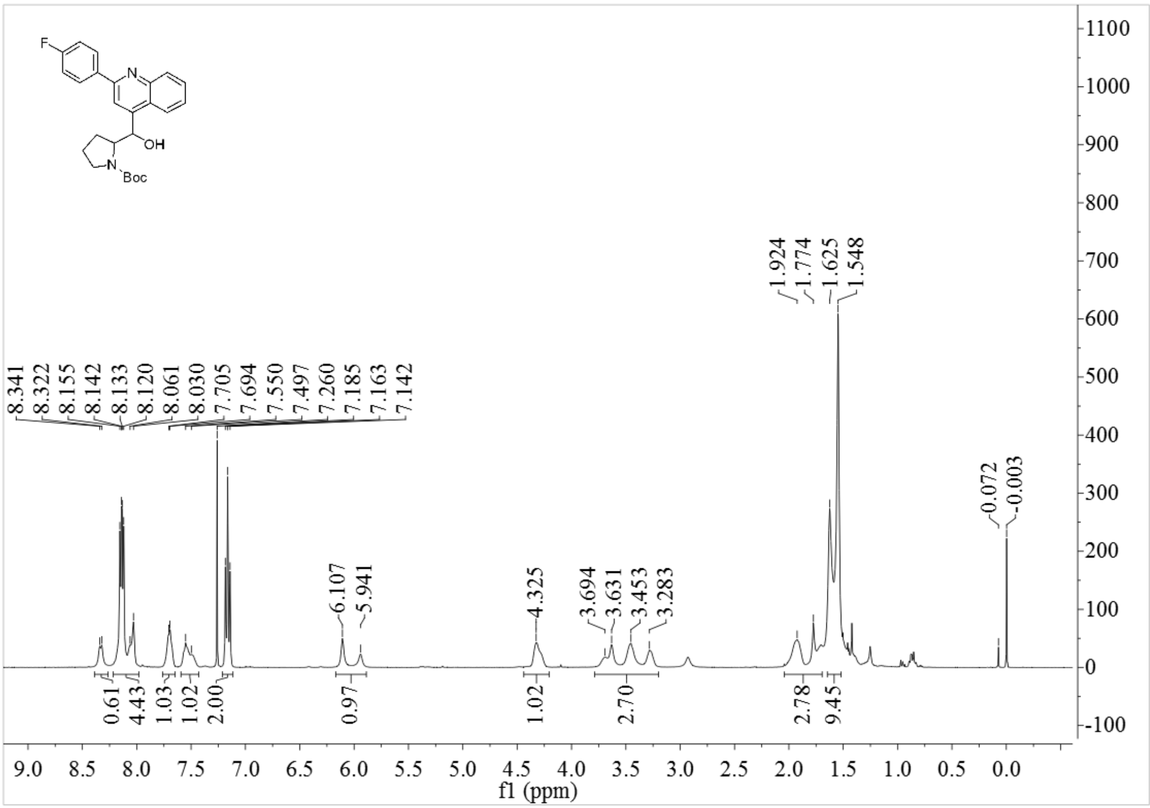

Fig S36. ^{1}H NMR spectrum of compound **II-6d.**

II-6d: 2-(Hydroxy(2-(4-fluorophethyl)quinolin-4-yl)methyl)pyrrolidine-1-carboxylic acid tert-butyl ester

White powder. ^{1}H NMR (400 MHz, $CDCl_3$) δ 8.34 (s, 1H), 8.16-8.03 (m, 4H), 7.71 (s, 1H), 8.13 (s, 1H), 7.70 (d, J = 4.4 Hz, 3H), 7.55-7.50 (m, 1H), 7.16 (t, J = 8.6 Hz, 1H), 6.08 (d, J = 66.4 Hz, 1H), 4.33 (s, 1H), 3.69-3.28 (m, 1H), 1.92-1.77 (m, 3H), 1.55 (s, 9H).

II-6d' (the more polar compound)

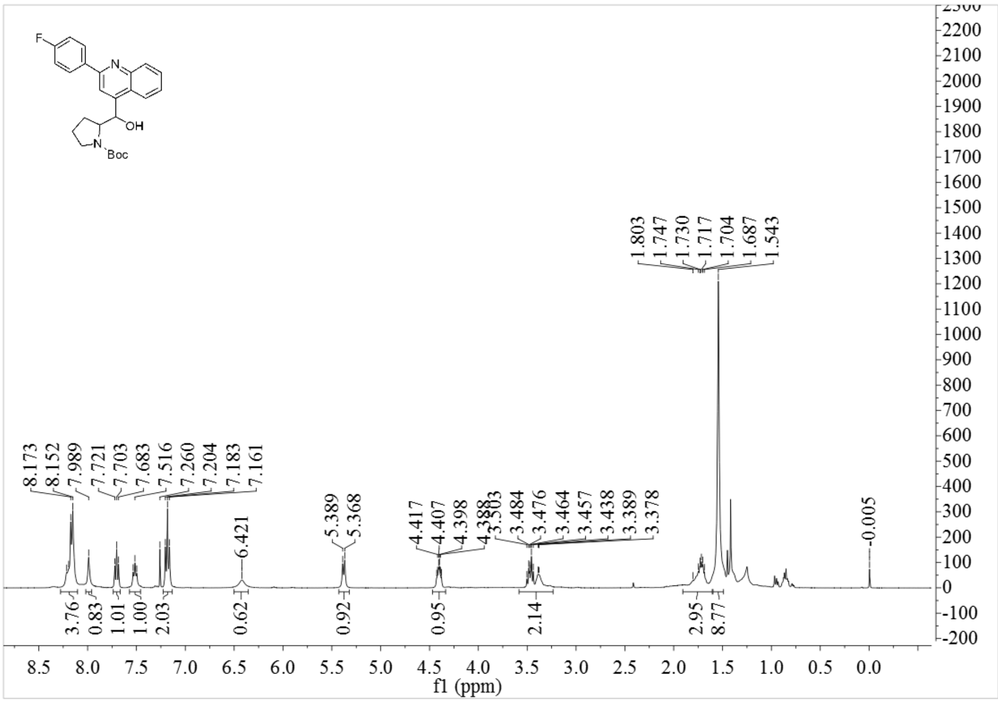

Fig S37. ^{1}H NMR spectrum of compound **II-6d′.**

II-6d′: 2-(Hydroxy(2-(4-fluorophethyl)quinolin-4-yl)methyl)pyrrolidine-1-carboxylic acid tert-butyl ester

White powder. ^{1}H NMR (400 MHz, $CDCl_3$) δ 8.18 (dd, J = 16.6, 8.4 Hz, 4H), 7.99 (s, 1H), 7.70 (t, J = 7.6 Hz, 1H), 7.52 (t, J = 7.4 Hz, 1H), 7.18 (t, J = 8.6 Hz, 2H), 6.42 (s, 1H), 5.38 (d, J = 8.2 Hz, 1H), 4.40 (td, J = 8.0, 3.8 Hz, 1H), 3.45 (ddd, J = 31.4, 17.4, 6.0 Hz, 2H), 1.90 – 1.62 (m, 3H), 1.54 (s, 9H).

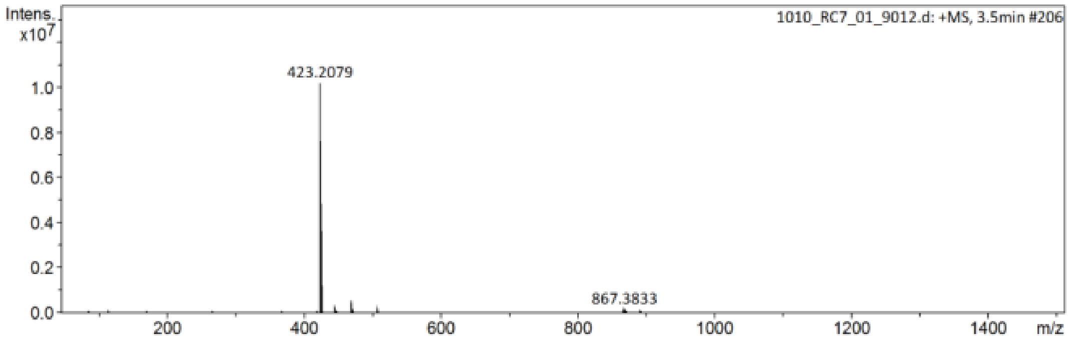

Fig S38. HRMS (ESI) spectrum of compound **II-6d.**

II-6d: 2-(Hydroxy(2-(4-fluorophethyl)quinolin-4-yl)methyl)pyrrolidine-1-carboxylic acid tert-butyl ester

HRMS (ESI), m/z calcd. For $C_{25}H_{28}FN_2O_3^+$ ([M+H]+) 423.2078, found: 423.2079.

II-7d

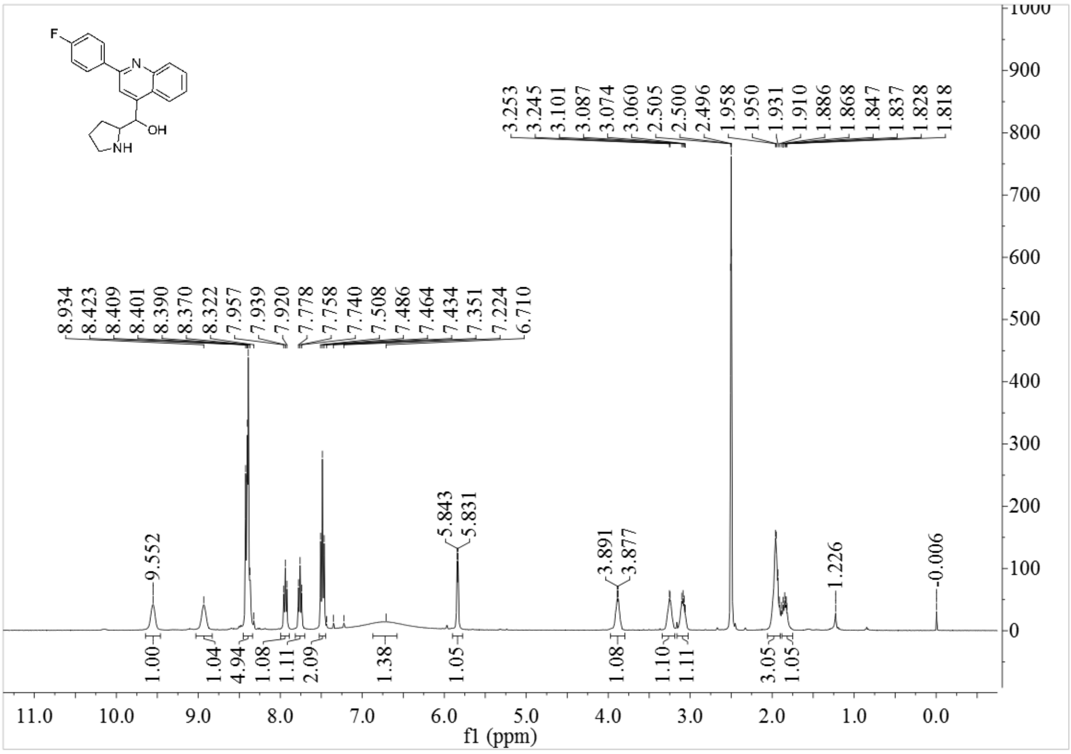

Fig S39. ^{1}H NMR spectrum of compound **II-7d.**

II-7d: (2-(4-fluorophethyl)quinolin-4-yl)(pyrrolidin-2-yl)methanol

White powder; m.p., 176-178 ºC; ^{1}H NMR (400 MHz, DMSO) δ 9.55 (s, 1H), 8.93 (s, 1H), 8.50 – 8.32 (m, 5H), 7.94 (t, J = 7.6 Hz, 1H), 7.76 (t, J = 7.6 Hz, 1H), 7.60 – 7.43 (m, 2H), 6.71 (s, 1H), 5.84 (d, J = 4.8 Hz, 1H), 5.84 (d, J = 4.8 Hz, 1H), 3.88 (d, J = 5.6 Hz, 1H), 3.25 (d, J = 3.4 Hz, 1H), 3.08 (dd, J = 10.8, 5.6 Hz, 1H), 2.05–1.91 (m, 3H), 1.91 – 1.80 (m, 1H).

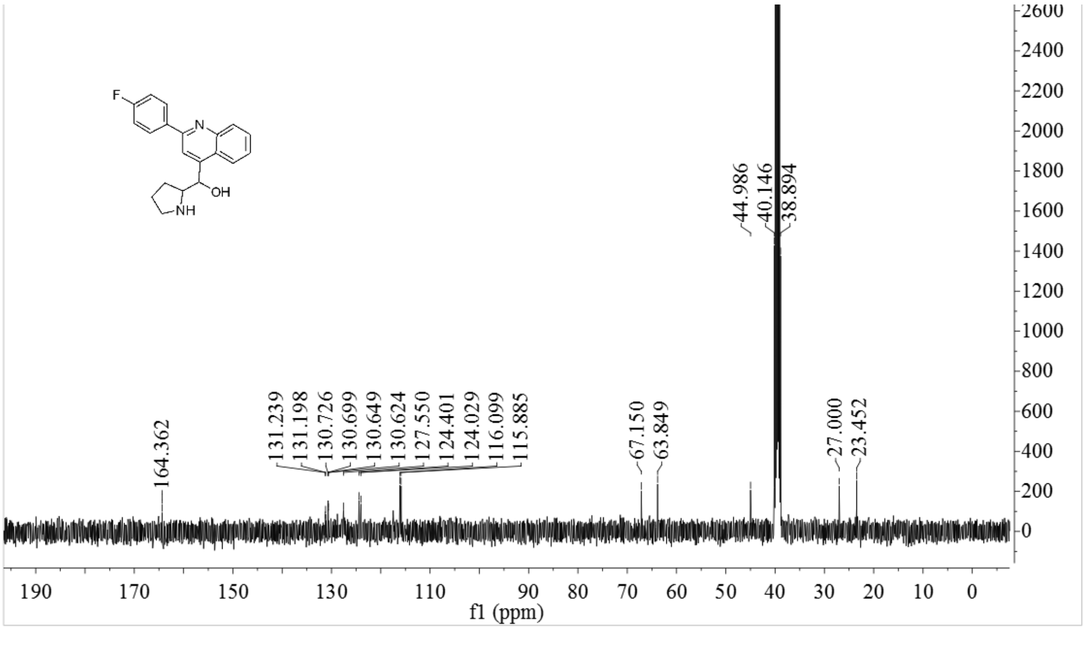

Fig S40. ^{13}C NMR spectrum of compound **II-7d.**

II-7d′: (2-(4-fluorophethyl)quinolin-4-yl)(pyrrolidin-2-yl)methanol

^{13}C NMR (101 MHz, DMSO) δ 164.4, 131.2, 131.2, 130.7, 130.7, 130.6, 130.6, 127.6, 124.4, 124.0, 117.5, 116.1, 115.9, 67.2, 63.85, 45.0, 27.0, 23.4.

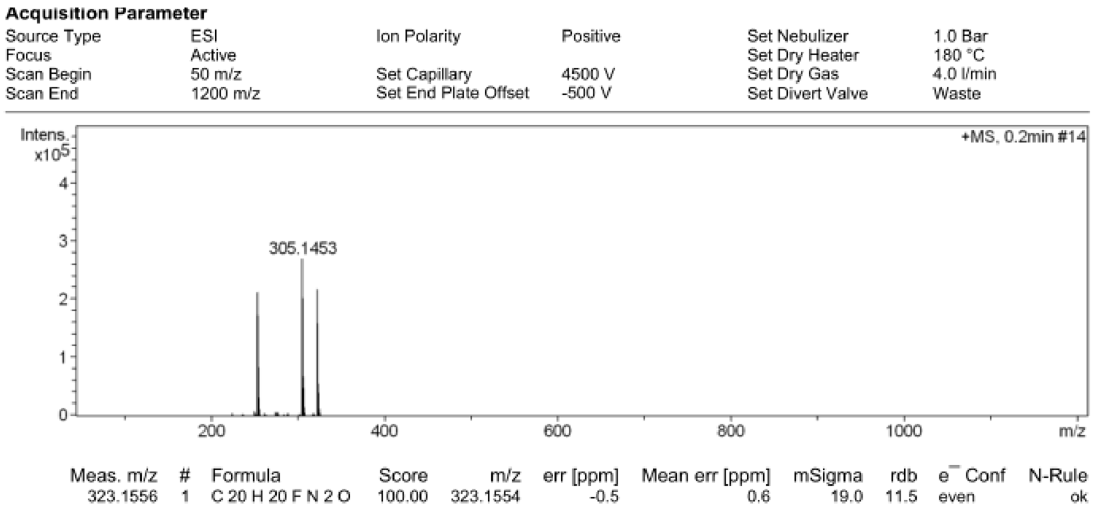

Fig S41. HRMS (ESI) spectrum of compound **II-7d.**

II-7d: (2-(4-fluorophethyl)quinolin-4-yl)(pyrrolidin-2-yl)methanol

HRMS (ESI), *m/z* calcd. For $C_{20}H_{20}FN_2O$([M+H]$^+$) 323.1554, found: 323.1556.

YS-4a

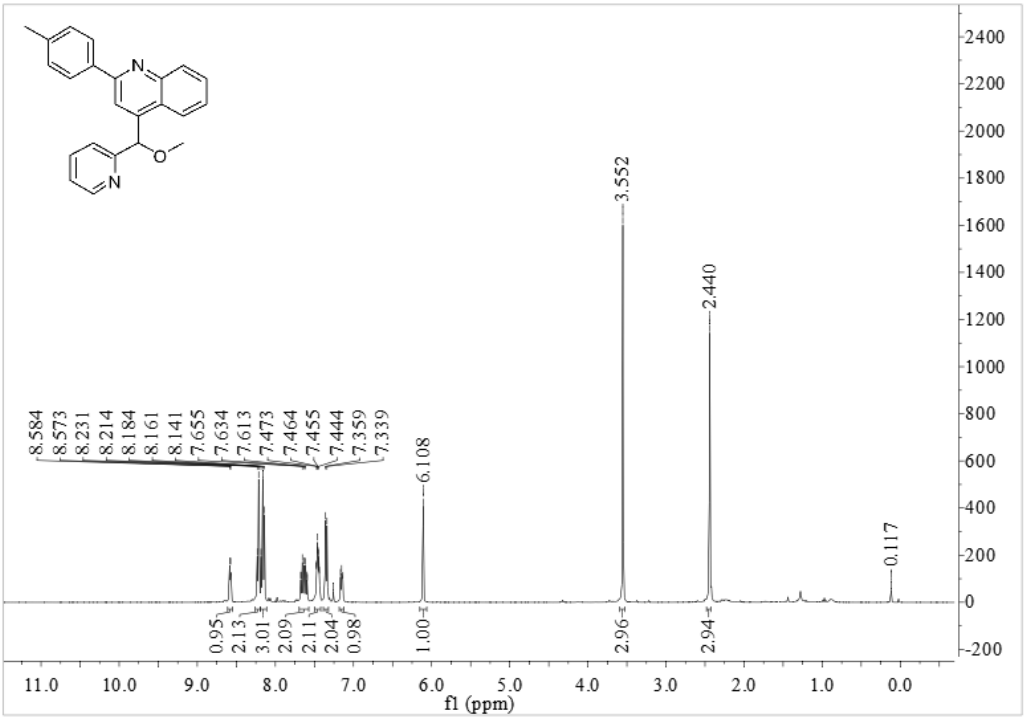

Fig S42. ^{1}H NMR spectrum of compound **YS-4a.**

YS-4a: 4-(methoxy(pyridin-2-yl)methyl)-2-(p-tolyl)quinoline

White powder. ^{1}H NMR (400 MHz, $CDCl_3$) δ 8.58 (d, J = 4.3 Hz, 1H), 8.22 (d, J = 6.8 Hz, 2H), 8.16 (t, J = 8.6 Hz, 3H), 7.71 – 7.55 (m, 2H), 7.49 – 7.43 (m, 2H), 7.35 (d, J = 8.0 Hz, 2H), 7.20 – 7.10 (m, 1H), 6.11 (s, 1H), 3.55 (s, 3H), 2.44 (s, 3H).

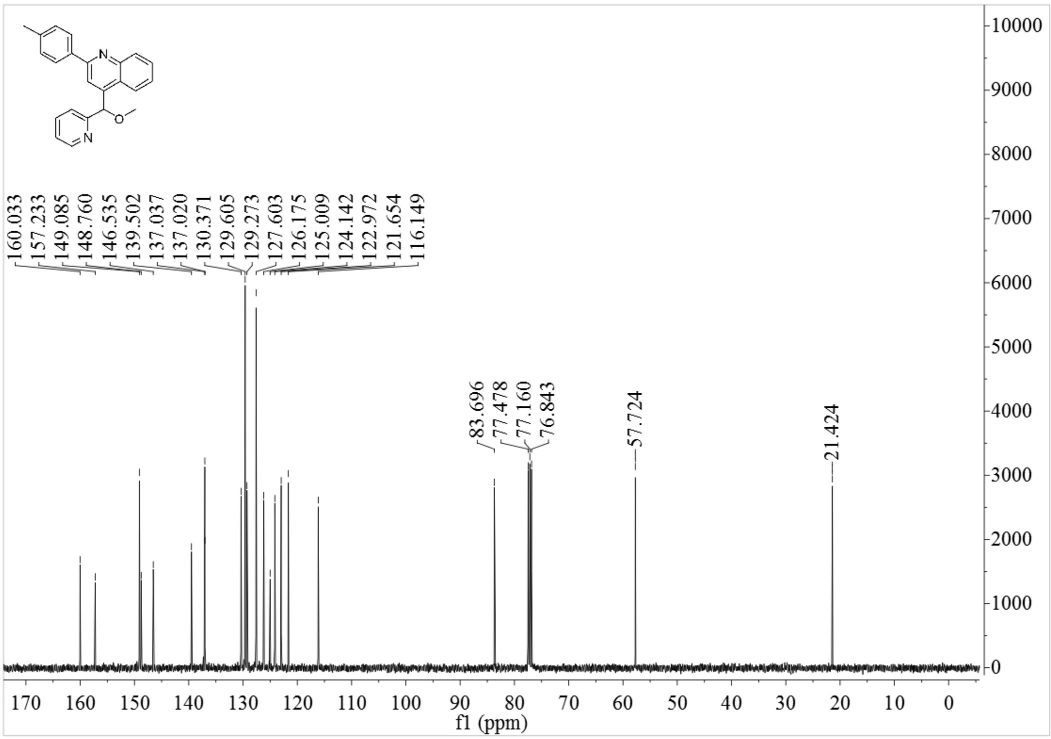

Fig S43. ^{13}C NMR spectrum of compound **YS-4a.**

YS-4a: 4-(methoxy(pyridin-2-yl)methyl)-2-(p-tolyl)quinoline

^{13}C NMR (101 MHz, $CDCl_3$) δ 160.0, 157.2, 149.1, 148.8, 146.5, 139.5, 137.0, 137.0, 130.4, 129.6, 129.3, 127.6, 126.2, 125.0, 124.1, 123.0, 121.6, 116.2, 83.7, 57.7, 21.4.

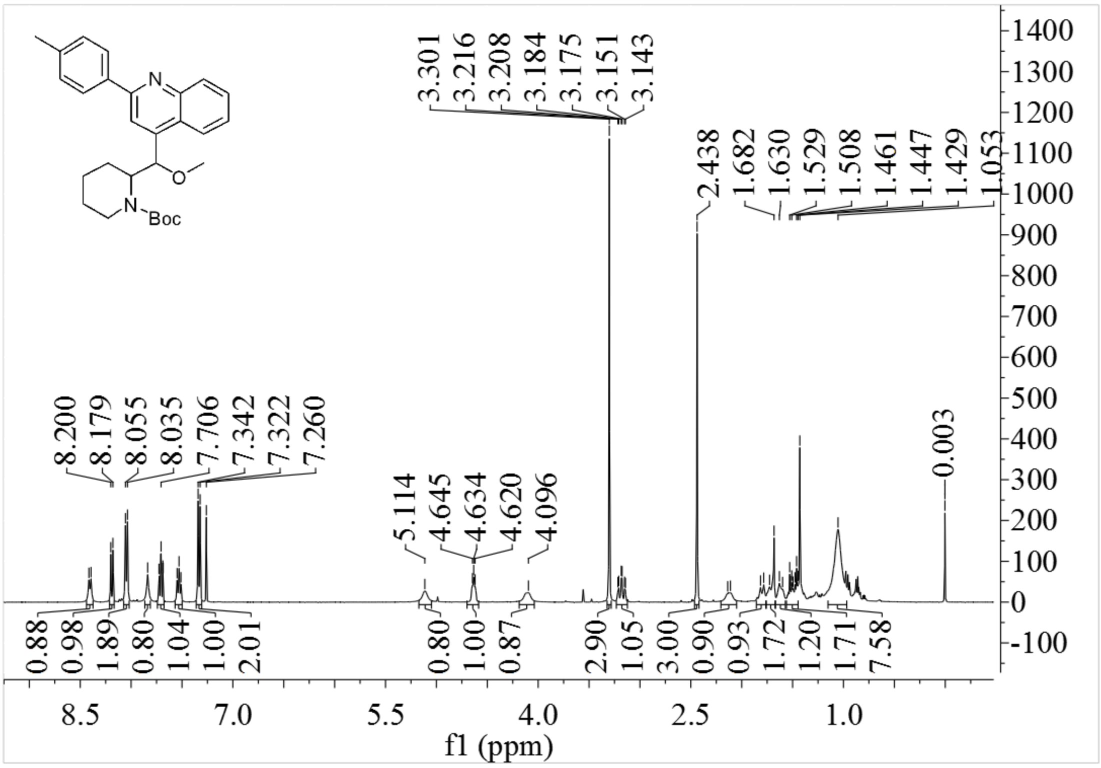

Fig S44. ^{1}H NMR spectrum of compound **YS-6a.**

YS-6a: Methoxy (2-(p-tolyl)quinolin-4-yl)methyl)piperidine-1-carboxylic acid tert-butyl ester

White solid. ^{1}H NMR (400 MHz, CDCl3) δ 8.40 (d, J = 8.2 Hz, 1H), 8.19 (d, J = 8.4 Hz, 1H), 8.05 (d, J = 8.0 Hz, 2H), 7.84 (s, 1H), 7.71 (ddd, J = 8.4, 6.9, 1.2 Hz, 1H), 7.53 (t, J = 7.5 Hz, 1H), 7.33 (d, J = 8.0 Hz, 2H), 5.11 (s, 1H), 4.63 (t, J = 5.0 Hz, 1H), 4.10 (s, 1H), 3.30 (s, 3H), 3.18 (td, J = 13.2, 3.2 Hz, 1H), 2.44 (s, 3H), 2.12 (d, J = 10.8 Hz, 1H), 1.80 (d, J = 12.4 Hz, 1H), 1.70 (d, J = 17.6 Hz, 2H), 1.61 (d, J = 12.6 Hz, 1H), 1.56-1.45 (m, 2H), 1.05 (s, 8H).

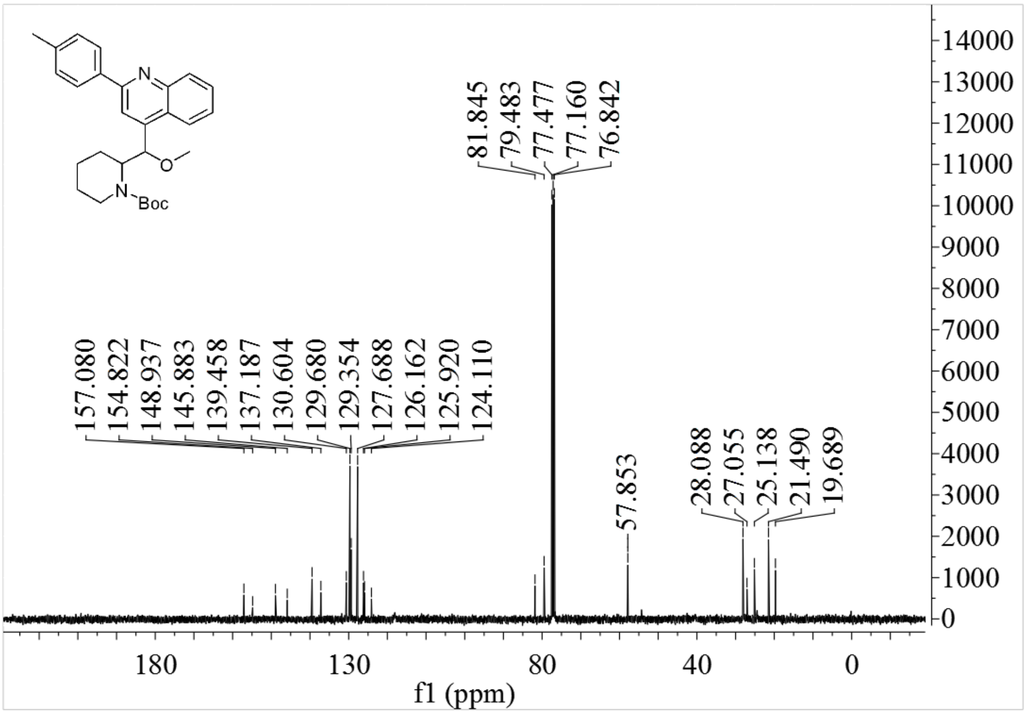

Fig S45. ^{13}C NMR spectrum of compound **YS-6a.**

YS-6a: Methoxy (2-(p-tolyl)quinolin-4-yl)methyl)piperidine-1-carboxylic acid tert-butyl ester

^{13}C NMR (101 MHz, CDCl3) δ 157.1, 154.8, 148.9, 145.9, 139.5, 137.2, 130.6, 129.7, 129.4, 127.7, 126.2, 125.9, 124.1, 81.8, 79.5, 57.8, 28.1, 27.0, 25.1, 21.5, 19.7.

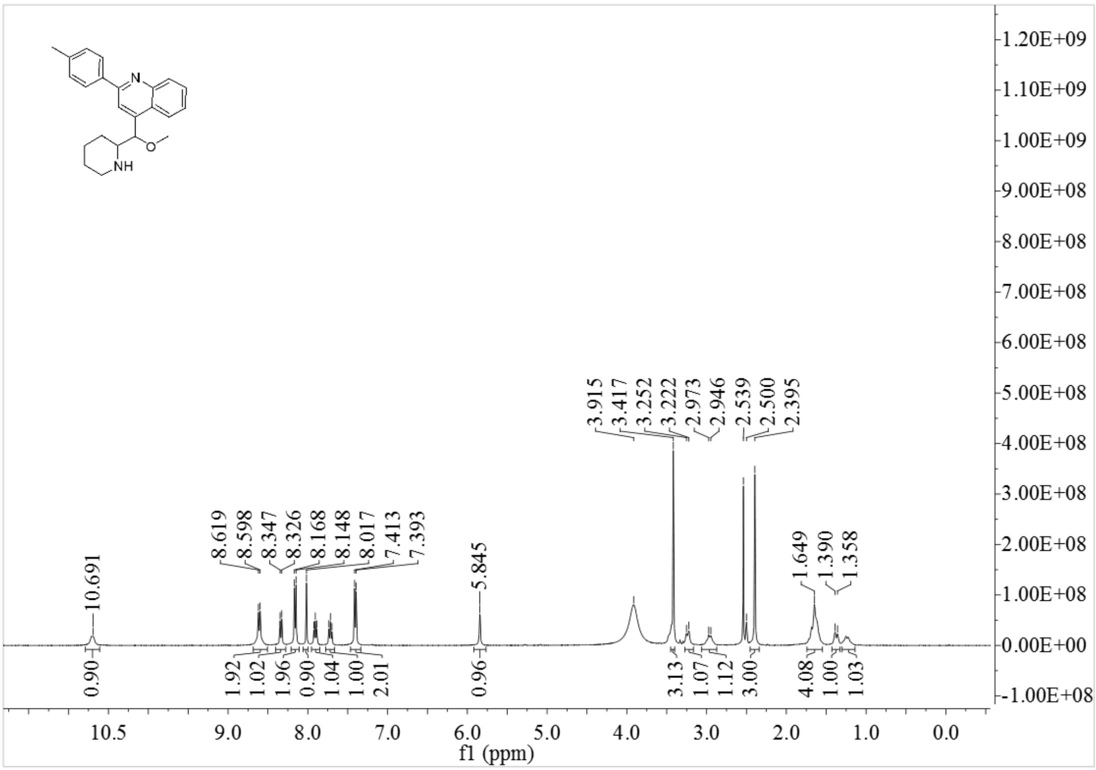

Fig S46. ^{1}H NMR spectrum of compound **YS-7a.**

YS-7a: 4-(Methoxy(piperidin-2-yl)methyl)-2-(p-tolyl)quinoline

Yellow solid; m.p., 134-136 ºC. ^{1}H NMR (400 MHz, DMSO) δ 10.69 (s, 1H), 8.61 (d, J = 8.4 Hz, 1H), 8.34 (d, J = 8.4 Hz, 1H), 8.16 (d, J = 8.0 Hz, 1H), 8.02 (s, 1H), 7.91 (t, J = 7.6 Hz, 1H), 7.72 (t, J = 7.6 Hz, 1H), 7.40 (d, J = 8.0 Hz, 1H), 5.84 (s, 1H), 3.42 (s, 2H), 3.24 (d, J = 11.8 Hz, 1H), 2.96 (d, J = 10.8 Hz, 1H), 2.54 (s, 1H), 2.39 (s, 2H), 1.65 (t, J = 11.6 Hz, 2H), 1.37 (d, J = 12.6 Hz, 1H), 1.24 (d, J = 12.2 Hz, 1H).

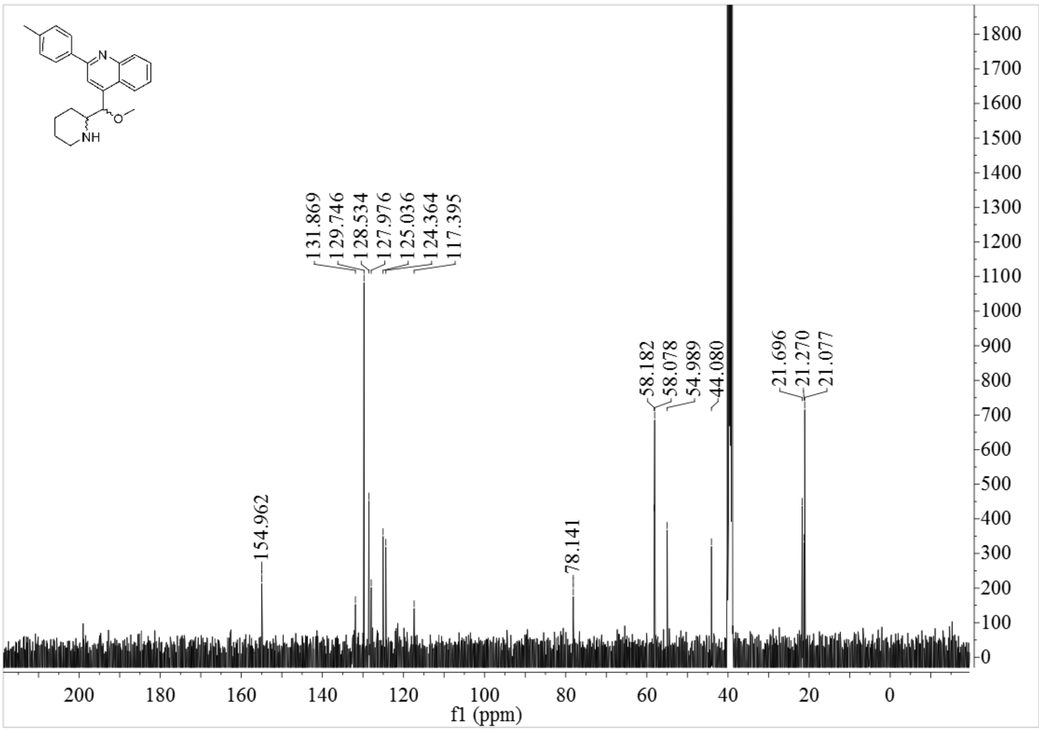

Fig 47. ^{13}C NMR spectrum of compound **YS-7a.**

YS-7a: 4-(Methoxy(piperidin-2-yl)methyl)-2-(p-tolyl)quinoline

^{13}C NMR (101 MHz, DMSO) δ 155.0, 131.9, 129.8, 128.5, 128.0, 125.0, 124.4, 117.4, 78.1, 58.2, 58.1, 55.0, 44.1, 21.7, 21.3, 21.1.

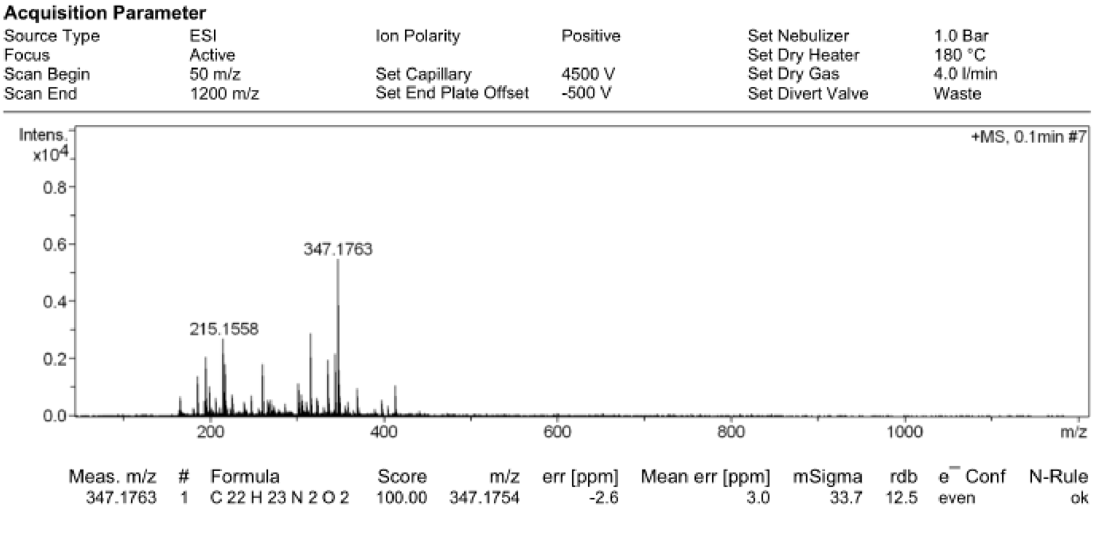

Fig S48. HRMS (ESI) spectrum of compound **YS-7a.**

YS-7a: 4-(Methoxy(piperidin-2-yl)methyl)-2-(p-tolyl)quinoline

HRMS (ESI): Exact Mass for C23H26N2O [M + H] + requires m/z 347.2118, found m/z 347.2118.

YS-4b

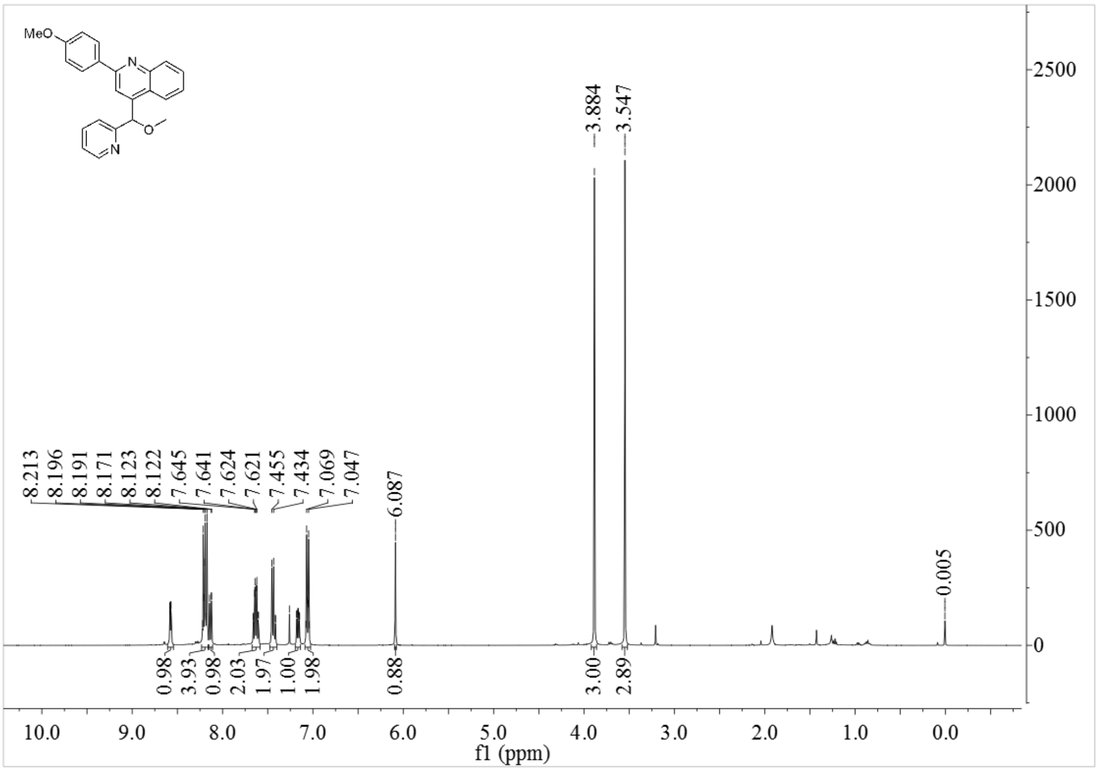

Fig S49. ^{1}H NMR spectrum of compound **YS-4b.**

YS-4b: 4-(Methoxy(pyridin-2-yl)methyl)-2-(4-methoxyphenyl)quinoline

White solid. ^{1}H NMR (600 MHz, $CDCl_3$) δ 8.58 (d, J = 4.8 Hz, 1H), 8.20 (d, J = 9.0 Hz, 2H), 8.17 (d, J = 6.0 Hz, 2H), 8.13 (d, J = 8.4 Hz, 1H), 7.64 (dd, J = 15.0, 7.2 Hz, 2H), 7.44 (t, J = 7.8 Hz, 2H), 7.18 (dd, J = 7.2, 4.8 Hz, 1H), 7.06 (d, J = 9.0 Hz, 2H), 6.08 (s, 1H), 3.90 (s, 3H), 3.55 (s, 3H).

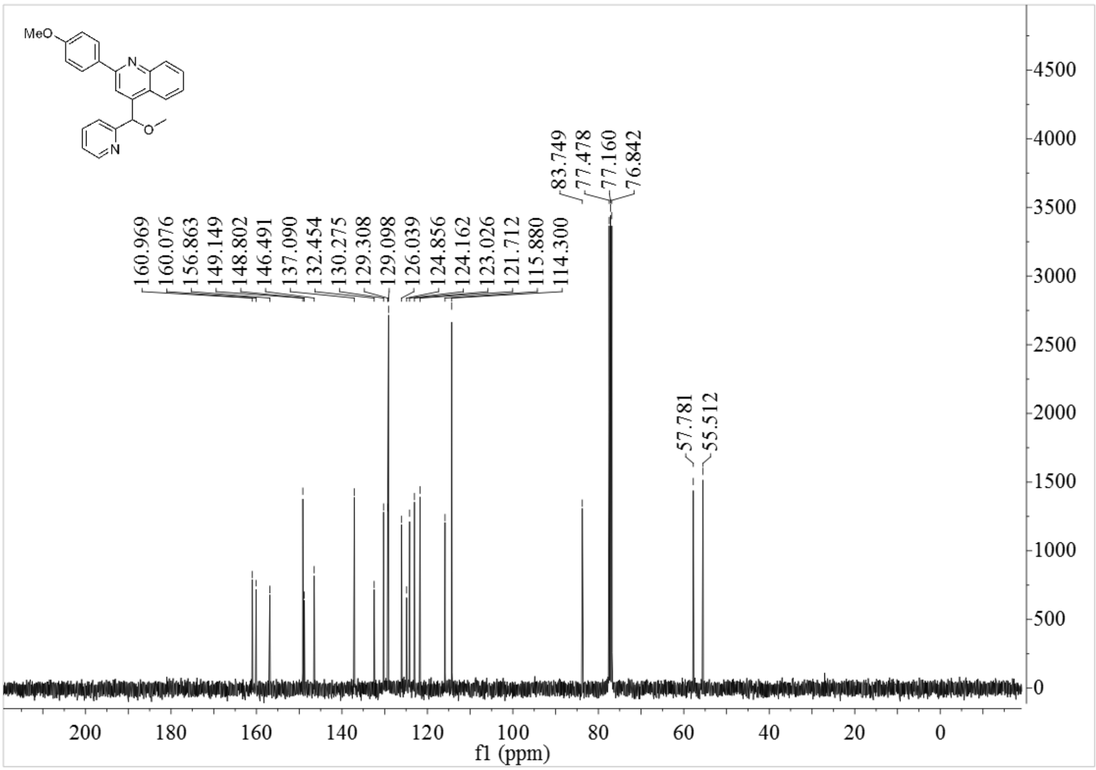

Fig S50. ^{13}C NMR spectrum of compound **YS-4b.**

YS-4b:: 4-(Methoxy(pyridin-2-yl)methyl)-2-(4-methoxyphenyl)quinoline

^{13}C NMR (151 MHz, $CDCl_3$) δ 160.9, 16., 156.8, 149.1, 148.7, 146.4, 137.0, 132.4, 130.2, 129.2, 129.0, 124.8, 124.1, 123.0, 121.6, 115.8, 114.2, 83.6, 57.7, 55.4..

YS-6b

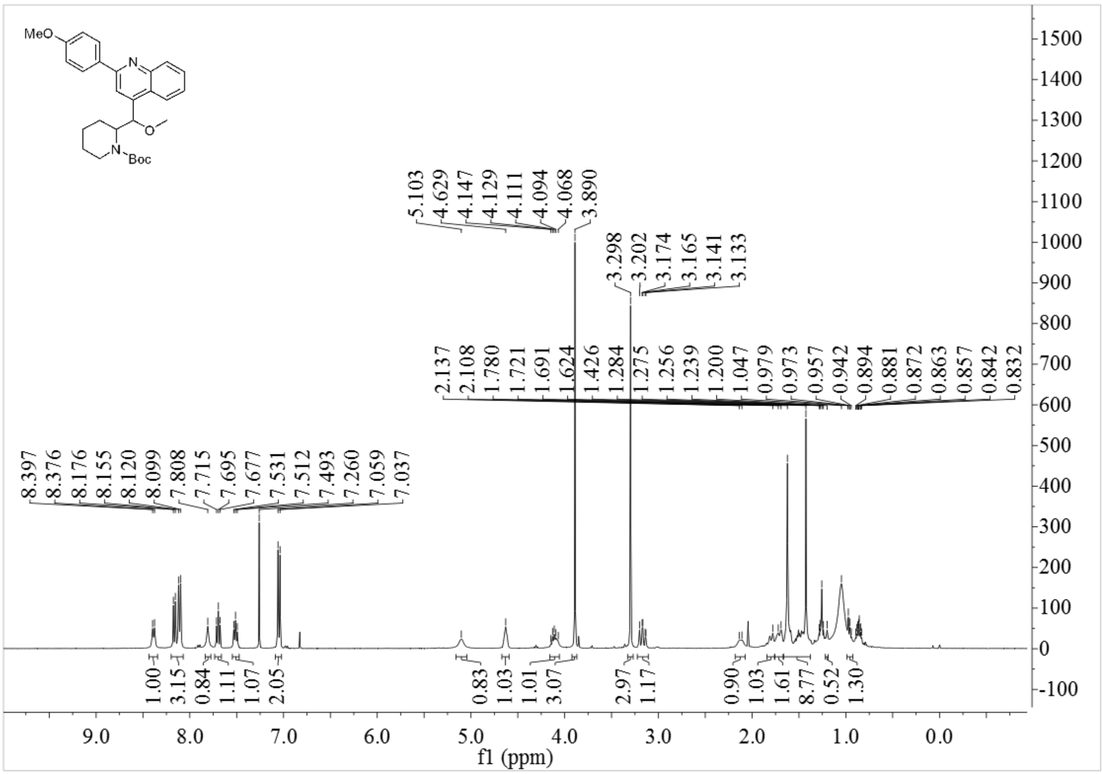

Fig S51. ^{1}H NMR spectrum of compound **YS-6b.**

YS-6b: (Methoxy (2-(4-methoxyphenyl)quinolin-4-yl)methyl)piperidine-1-carboxylic acid tert-butyl ester

White solid. ^{1}H NMR (400 MHz, $CDCl_3$) δ 8.39 (d, J = 8.4 Hz, 1H), 8.17 (d, J = 8.4 Hz, 1H), 8.11 (d, J = 8.6 Hz, 2H), 7.81 (s, 1H), 7.70 (t, J = 7.6 Hz, 1H), 7.51 (t, J = 7.6 Hz, 1H), 7.05 (d, J = 8.8 Hz, 2H), 5.10 (s, 1H), 4.63 (s, 1H), 4.11 (dt, J = 17.4, 8.6 Hz, 1H), 3.89 (s, 3H), 3.30 (s, 3H), 3.25 – 3.10 (m, 1H), 2.12 (d, J = 11.6 Hz, 1H), 1.80 (d, J = 12.7 Hz, 1H), 1.78 (s, 1H), 1.71 (d, J = 11.9 Hz, 1H), 1.62 (s, 9H), 1.20 (s, 1H), 0.96 (dd, J = 10.8, 4.2 Hz, 1H).

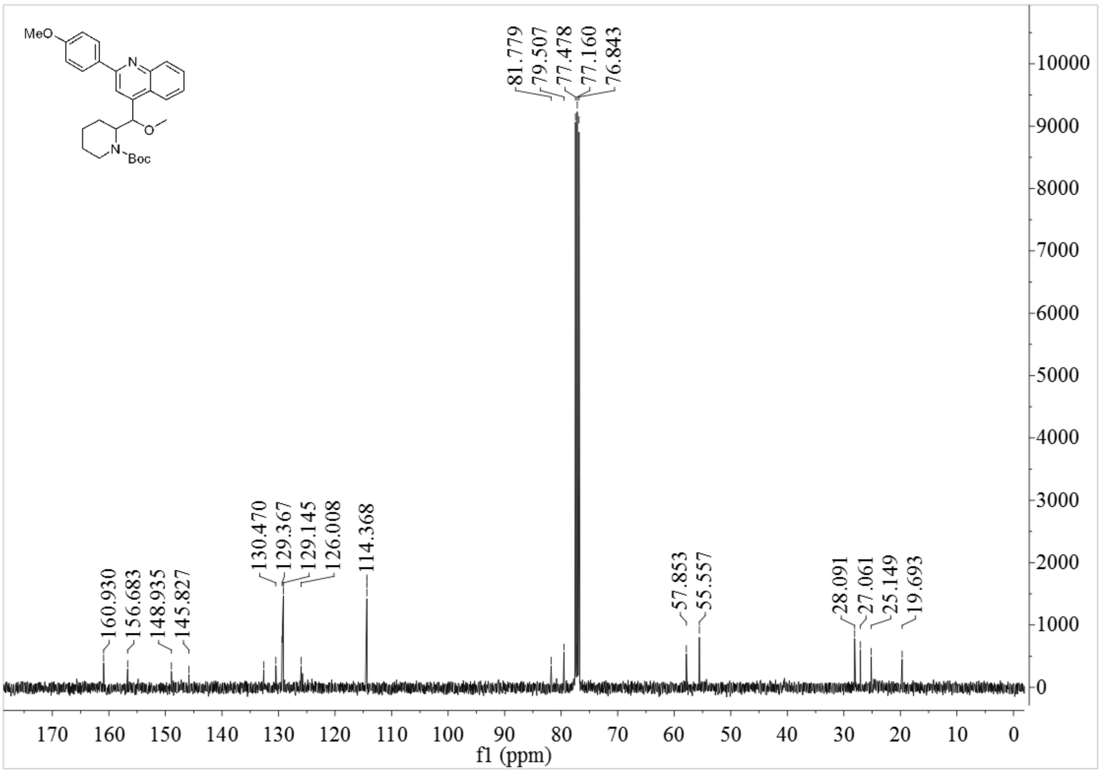

Fig S52. ^{13}C NMR spectrum of compound **YS-6b.**

YS-6b: (Methoxy (2-(4-methoxyphenyl)quinolin-4-yl)methyl)piperidine-1-carboxylic acid tert-butyl ester

^{13}C NMR (101 MHz, $CDCl_3$) δ 160.9, 156.7, 148.9, 145.8, 132.6, 130.5, 129.4, 129.2, 126.0, 114.4, 81.8, 79.5, 57.8, 55.6, 28.1, 27.1, 25.2, 19.7.

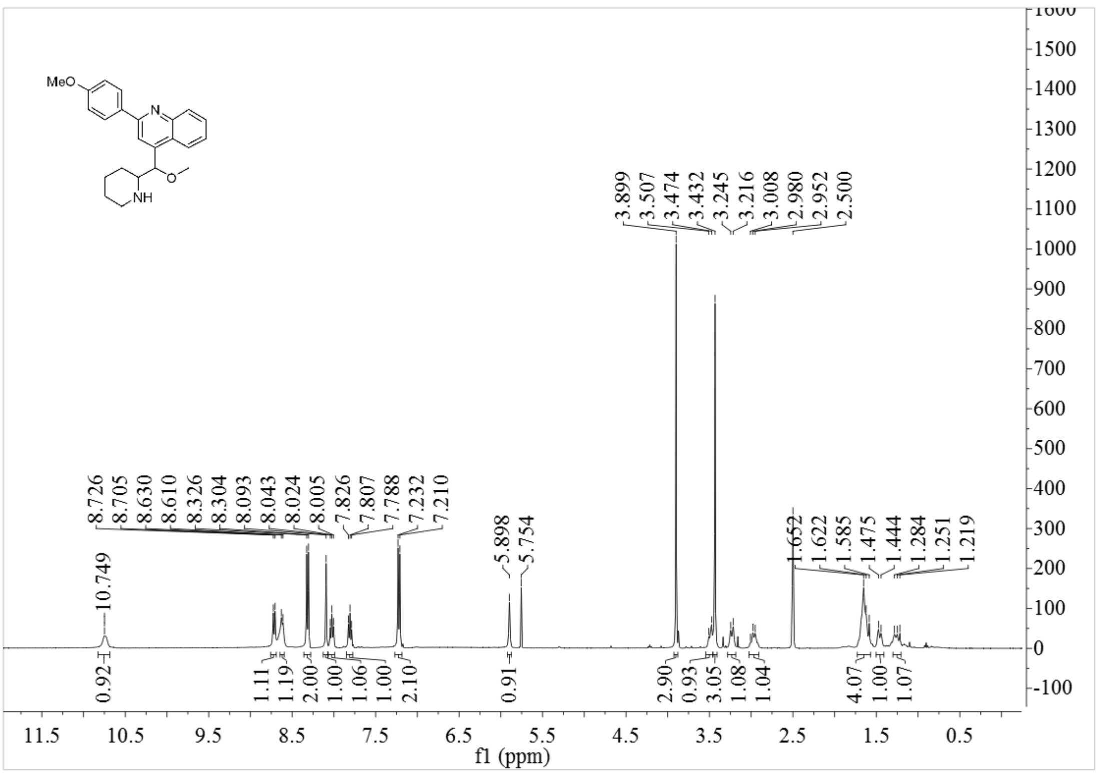

Fig S53. ^{1}H NMR spectrum of compound **YS-7b.**

YS-7b: 4-(Methoxy(piperidin-2-yl)methyl)-2-(4-methoxyphenyl)quinoline

Yellow solid; m.p., 177-179 °C. ^{1}H NMR (400 MHz, DMSO) δ 10.75 (s, 1H), 8.72 (d, J = 8.4 Hz, 1H), 8.62 (d, J = 8.0 Hz, 1H), 8.32 (d, J = 8.8 Hz, 2H), 8.09 (s, 1H), 8.02 (t, J = 7.6 Hz, 1H), 7.81 (t, J = 7.6 Hz, 1H), 7.22 (d, J = 8.8 Hz, 2H), 5.90 (s, 1H), 3.90 (s, 3H), 3.49 (d, J = 13.2 Hz, 1H), 3.35 (s, 3H), 3.23 (d, J = 11.8 Hz, 1H), 3.02 – 2.87 (m, 1H), 1.69 – 1.53 (m, 4H), 1.46 (d, J = 12.2 Hz, 1H), 1.31 – 1.16 (m, 1H).

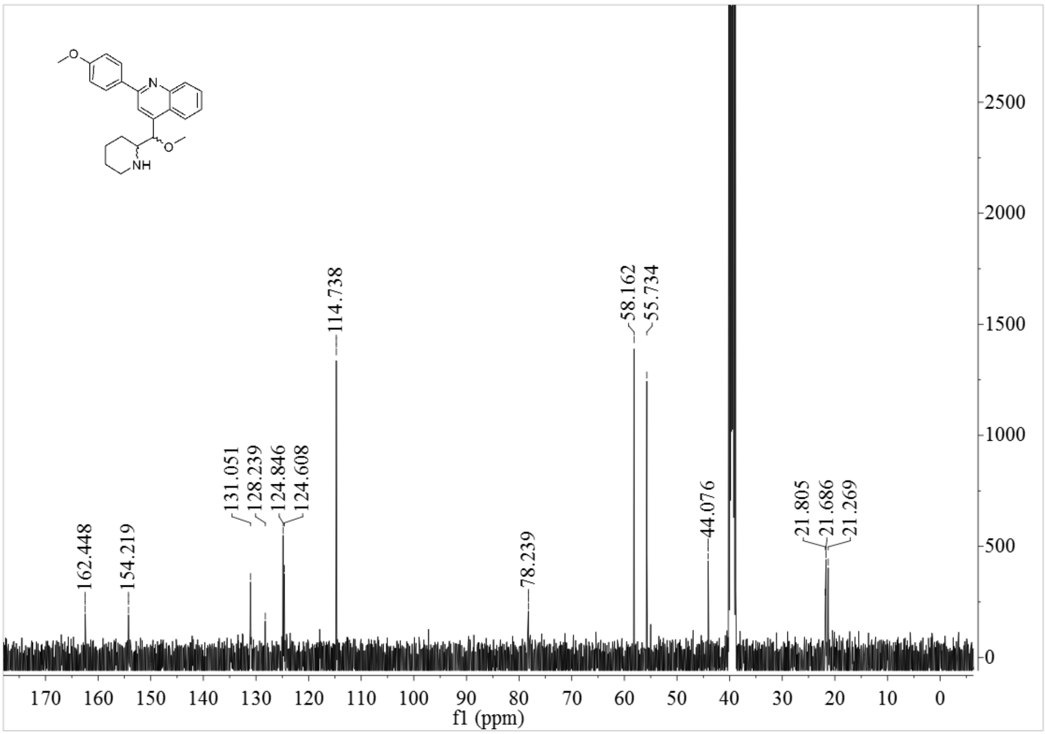

Fig S54. ^{13}C NMR spectrum of compound **YS-7b.**

YS-7b: 4-(Methoxy(piperidin-2-yl)methyl)-2-(4-methoxyphenyl)quinoline

^{13}C NMR (101 MHz, DMSO) δ 162.4, 154.2, 131.1, 128.2, 124.6, 114.7, 78.2, 58.2, 55.7, 44.1, 40.1, 39.9, 39.7, 39.5, 39.3, 39.1, 38.9, 21.8, 21.7, 21.3.

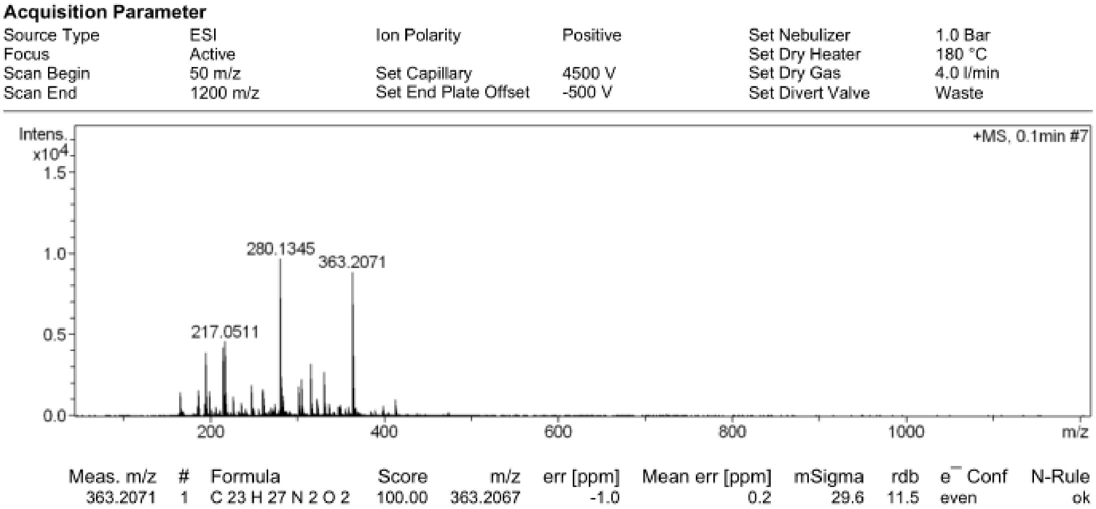

Fig S55. HRMS (ESI) spectrum of compound **YS-7b.**

YS-7b: 4-(Methoxy(piperidin-2-yl)methyl)-2-(4-methoxyphenyl)quinoline

HRMS (ESI): Exact Mass for C23H26N2O2 [M + H] + requires m/z 363.2067, found m/z 363.2071.
